# Secreted Protein Acidic and Rich in Cysteine (*Sparc*) KO Leads to an Accelerated Ageing Phenotype Which Is Improved by Exercise Whereas SPARC Overexpression Mimics Exercise Effects in Mice

**DOI:** 10.3390/metabo12020125

**Published:** 2022-01-28

**Authors:** Abdelaziz Ghanemi, Aicha Melouane, Mayumi Yoshioka, Jonny St-Amand

**Affiliations:** 1 Functional Genomics Laboratory, Endocrinology and Nephrology Axis, CHU de Québec-Université Laval Research Center, Québec, QC G1V 4G2, Canada; abdelaziz.ghanemi@crchudequebec.ulaval.ca (A.G.); aicha.melouane@crchudequebec.ulaval.ca (A.M.); mayumi.yoshioka@crchudequebec.ulaval.ca (M.Y.); 2 Department of Molecular Medicine, Faculty of Medicine, Laval University, Québec, QC G1V 0A6, Canada

**Keywords:** secreted protein acidic and rich in cysteine (SPARC), exercise, ageing, metabolism

## Abstract

Secreted protein acidic and rich in cysteine (SPARC) is a matricellular glycoprotein implicated in various functions, including metabolism, tissue regeneration, and functional homeostasis. SPARC/*Sparc* declines with ageing but increases with exercise. We aim to verify two hypotheses: (1) SPARC deficiency leads to an ageing-like phenotype (metabolic decline, muscle loss, etc.), and (2) SPARC overexpression would mimic exercise, counteract ageing, and improve age-related changes. Our mice experiments are divided into two parts. First, we explore the consequences of *Sparc* knockout (KO) and compare them to the ageing effects. We also observe the effects of exercise. In the second part, we study the effects of SPARC overexpression and compare them to the exercise benefits. At the end, we make an analysis of the results to point out the analogies between *Sparc* KO and the ageing-like phenotype on the one hand and make comparisons between SPARC overexpression and exercise in the context of exercise counteracting ageing. The measurements were mainly related to tissue weights, adiposity, metabolism, and muscle strength. The main findings are that *Sparc* KO reduced glucose tolerance, muscle glucose transporter expression, and abdominal adipose tissue weight but increased glycogen content in the muscle. SPARC overexpression increased muscle strength, muscle mass, and expressions of the muscle glucose transporter and mitochondrial oxidative phosphorylation but lowered the glycemia and the adiposity, especially in males. Collectively, these findings, and the data we have previously reported, show that *Sparc* KO mice manifest an ageing-like phenotype, whereas SPARC overexpression and exercise generate similar benefits. The benefits are towards counteracting both the SPARC deficiency-induced ageing-like phenotype as well as reversing the age-related changes. The potential applications of these findings are to build/optimize *Sparc* KO-based animal models of various health conditions and, on the other hand, to develop therapies based on introducing SPARC or targeting SPARC-related pathways to mimic exercise against age-related and metabolic disorders.

## 1. Introduction

With the improvement of the healthcare system and the decline of infectious diseases (vaccines, therapies, etc.), life expectancy has increased significantly over the past 50 years [1], which has enhanced the elderly population. This makes ageing and age-associated metabolic and functional decline important health challenges as they represent risk factors for various health problems, including metabolic disorders and obesity. Geriatrics aims to develop the best medical approaches to face such challenges. Within this context, exercise is the best anti-ageing approach as it minimizes several age-related changes [2,3] and has been prescribed for the older population [4,5,6]. In addition, exercise has been medically prescribed for a variety of other health conditions, such as obesity and sarcopenia, for which ageing could be a risk factor as well. Therefore, elucidating the molecular pathways of both ageing and exercise and their mechanistic links would significantly contribute to optimizing both the available studying methods (animal models of ageing, cell cultures, etc.) and the available therapeutic tools. 

In order to better elucidate the exercise molecular patterns and the mechanism by which exercise leads to its known benefits, we have already characterized secreted protein acidic and rich in cysteine (*SPARC*) as an exercise-induced gene in vivo [7] and in vitro (cellular model of exercise) [8]. SPARC serum levels also increase with exercise [9], both in humans and in mice [10]. We have suggested that the exercise-induced muscle phenotype changes are SPARC-dependent [11], and exercise-induced *Sparc* expression could be an indicator of the therapeutic efficacy of exercise and the response to exercise [12]. In addition, adding SPARC to C2C12 (muscle cells) increased their differentiation [13]. On the other hand, *Sparc* expression is downregulated by ageing [10]. 

As *Sparc* decreases with ageing, and exercise both increases *Sparc/*SPARC [10,14] and counteracts ageing, we suggest that the ageing results and exercise benefits (including anti-ageing) are mediated, at least in part, by SPARC, especially in the way that age-related alterations in skeletal muscle progenitor cells are linked to SPARC, for instance [15]. These correlate with SPARC implications in different biological and metabolic functions. Indeed, SPARC, also known as osteonectin or basement membrane-40 (BM-40) [16,17], is a matricellular glycoprotein which comprises three distinct structural domains [18] and binds to calcium, collagen [19], and vitronectin (structural matrix proteins) [20]. It is expressed in most tissues, especially when the tissues undergo changes or active remodeling (healing, embryogenesis, cancer, obesity, etc.), which reflects its importance in tissue development and growth. To achieve such functions, SPARC is, for instance, involved in tissue repair [21], cell turnover [22], cell renewal and growth [23,24], maintenance of bone mass [25], osteoblast maturation [16], angiogenesis regulation [25], extracellular matrix organization [26], collagen maturation [27], glucose and lipid metabolism [28,29,30], remodeling [31,32], regeneration [33], differentiation [34], and adipose tissue regulation [35]. Such distribution and implications also allowed us to suggest SPARC as a molecular physiological and pathological biomarker [36].

Elucidating the interplay between ageing, SPARC, and exercise can represent a breakthrough for a deeper understanding of ageing and metabolic disorders towards novel SPARC-based molecular therapies for the related health conditions. Within this context, the roles and properties of SPARC allow us to formulate two hypotheses. Whereas SPARC deficiency leads to an ageing-like phenotype (metabolic decline, muscle loss, etc.), SPARC overexpression would mimic exercise, counteract ageing, and improve age-related changes (boost metabolism, strengthen muscles, reduce adiposity, etc.).

We have designed two independent, yet correlated, studies to verify our hypotheses. Our key molecular and functional measures focus on exploring skeletal muscle for specific reasons. First, the studies which revealed that *SPARC*/*Sparc* is overexpressed in response to exercise were conducted in muscles (in vivo) and muscle cell culture (in vitro). In addition, muscle is the main tissue implicated in the metabolic and mechanic functions and is modified by exercise; SPARC represents a myokine secreted by the muscle in response to exercise and represents a secretory organ. This is in addition to the role of muscles in generating force and power (mechanical property [37]). Indeed, the skeletal muscle implication in homeostasis is not limited to its metabolic activity (mainly energy expenditure); it is also an organ that secrets bioactive proteins, including interleukins and growth factors [9] which allow the muscle’s communication with diverse tissues [38], including key tissues such as the adipose tissue, the liver, and the brain [39]. Finally, the metabolic and contractile performance of the muscles changes with ageing (muscle weakness and atrophy [37]), which is a key variable that we focus on in this work. However, as adiposity and tissue weights are also important in the context of ageing and exercise, we have also added related measures to complete the investigation.

## 2. Results

### 2.1. Sparc Knockout (KO) Mice, Exercise, and Ageing

Each age group of the mice, young (Y) and old (O), was divided based on the genotypes of knockout (KO) or wild-type (WT) to obtain four groups: Y-KO, Y-WT, O-KO and O-WT. Finally, each of these four groups was further subdivided into two groups according to whether they were exercising (Ex) or sedentary (Sed) mice. Therefore, our experimental design included eight groups.

#### 2.1.1. Tissue Weights and Sizes

The mice tissues were weighed immediately after they were removed, following the sacrifice. The analyzed data (Table 1) are in the form of both the absolute values (weight) and the weight percentages of the tissues (to the body weight). The tissues were the Achilles tendon, brown adipose tissue (BAT), inguinal adipose tissue (IngAT), abdominal adipose tissue (AbdAT), retroperitoneal adipose tissue (RetAT), epididymal adipose tissue (Epi AT), and Mesenteric adipose tissue (MesAT) and the three muscles, gastrocnemius (GC), soleus (Sol), and extensor digitorum longus (EDL). We also provide inguinal and epididymal adipocytes size (measured) and number (estimated).

The data show a genotype effect (WT > KO) for all the tissues for either the weights or the weight percentages or for both, except for the BAT, for which we had an opposite genotype effect (KO > WT) for its weight percentage. In addition, a genotype effect (WT > KO) was seen on the estimated number of EpiAT adipocytes.

We found the effect of age (O > Y) on the weights of the Achilles tendon, BAT, IngAT, AbdAT, RetAT, EpiAT, and MesAT, as well as the weight percentages of the BAT (trends), AbdAT, RetAT, EpiAT, and MesAT, in addition to the adipocytes size of EpiAT and IngAT and the estimated adipocytes number of EpiAT. We had the opposite effect of age (Y > O) on the weights of GC and the weight percentages of GC, Sol, and EDL (trend), as well as the estimated adipocytes number of IngAT.

For the exercise effects, exercise increased the weight percentage of BAT but decreased the weights of AbdAT (trend), RetAT (trend), and MesAT and the weight percentage of MesAT.

The statistical analysis also revealed an interaction between genotype and age. Indeed, whereas the age effect (O > Y) was more pronounced in the WT mice than in the *Sparc* KO mice in the weight and weight percentages of AbdAT, RetAT, EpiAT and MesAT, it was only seen in the WT mice (not the *Sparc* KO mice) in the adipocytes size of IngAT and the estimated adipocytes number of EpiAT. On the other hand, only in the Achilles tendon was the age effect (O > Y) on weight more pronounced in the *Sparc* KO mice, and the age effect (O > Y) on weight percentage was only seen in the *Sparc* KO mice.

#### 2.1.2. Oral Glucose Tolerance Test (OGTT) 

**Figure 1:**
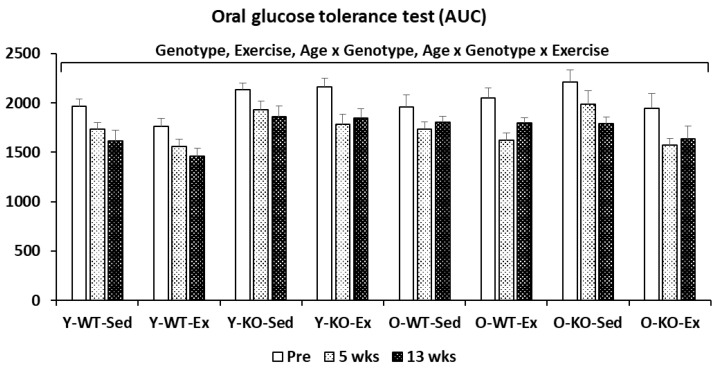
Oral Glucose Tolerance Test. Glucose tolerance decreased with *Sparc* KO and increased with exercise. The ageing also decreased the glucose tolerance only in WT mice (not in *Sparc* KO mice). The 4-way ANOVA analysis also revealed an interaction (age × genotype × exercise) in which ageing decreased the tolerance only in WT-Ex mice (not WT-Sed mice) and increased the glucose tolerance in KO-Ex mice (not KO-Sed mice). All data are mean ± SEM. The number of mice: 11–12 mice per experimental condition. Abbreviations: AUC, area under the curve; Ex, exercise; KO, knockout; O, old; Sed, sedentary; *Sparc*, secreted protein acidic and rich in cysteine; WT, wild-type; Y, young.

Figure 1 shows that the glucose tolerance decreased with the *Sparc* KO and increased with exercise. The ageing also decreased the glucose tolerance only in the WT mice (not in the *Sparc* KO mice). The 4-way ANOVA analysis also revealed an interaction (age × genotype × exercise) in which ageing decreased the tolerance only in the WT-Ex mice (not the WT-Sed mice) and increased the glucose tolerance in the KO-Ex mice (not the KO-Sed mice).

#### 2.1.3. Glycogen Content and Glucose Transporter Type 4 (GLUT4) Expression in the Muscle

**Figure 2:**
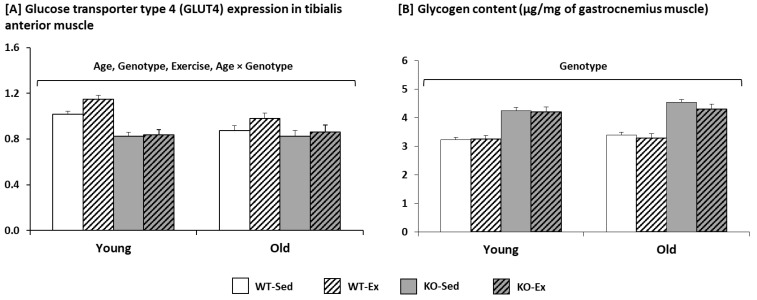
Glucose transporter type 4 (GLUT4) expression and glycogen content in the muscle. (**A**) Ageing and *Sparc* KO decreased muscular expression of GLUT4, but exercise increased it. The interaction (age × genotype) revealed that the effect of ageing was only seen in WT mice. (**B**) *Sparc* KO increased the muscular glycogen. All data are mean ± SEM. The number of mice: 11–12 mice per experimental condition. Abbreviations: Ex, exercise; KO, knockout; Sed, sedentary; *Sparc*, secreted protein acidic and rich in cysteine; WT, wild-type.

For the muscular expression of GLUT4 (Figure 2A), ageing and the *Sparc* KO decreased it, but exercise increased it. The interaction (age × genotype) revealed that the ageing effect was only seen in the WT mice.

Among the three variables, only the genotype had an effect on the glycogen content (Figure 2B). The *Sparc* KO increased the muscular glycogen.

### 2.2. Sparc Transgeneic (Tg) Mice

The study involved 32 mice. They were divided into four groups depending on the genotype, Tg (*Sparc* overexpression) or WT, and sex, male (M) or female (F). Therefore, we had four groups: Tg-M (*n* = 9), WT-M (*n* = 7), Tg-F (*n* = 11), and WT-F (*n* = 5)

#### 2.2.1. Tissues Weights

**Figure 3:**
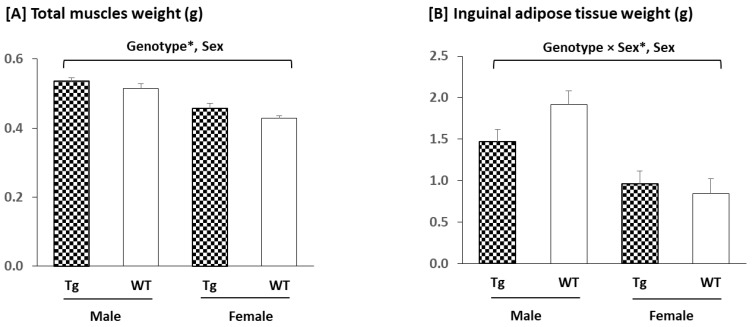
Total muscle and inguinal adipose tissue weights: (**A**) *Sparc* overexpression tended to increase the total skeletal muscles (gastrocnemius, soleus, tibialis anterior, and extensor digitorum longus) weight. Male mice had a heavier muscle weight than female mice. For the inguinal adipose tissue (**B**), the *Sparc* overexpression decreased its weight in male mice by 23%. Male mice had more inguinal adipose tissue than female mice. All data are mean ± SEM. The number of mice: 5–11 mice per experimental condition. *: Trend (0.05 ≤ *p* ≤ 0.1). Abbreviations: g, gram; *Sparc*, secreted protein acidic and rich in cysteine; Tg, transgenic (*Sparc* overexpression); WT, wild-type.

The mice overexpressing *Sparc* tended to have larger total skeletal muscle mass (GC, Sol, TA, and EDL) (Figure 3A). In addition, the muscle mass was greater in the male mice than in the female mice. For the IngAT mass (Figure 3B), the decreased (trend) weight was only seen in the male mice (by 23%), whereas the male mice had more IngAT than the female mice.

#### 2.2.2. Muscle Strength (Grip Power Test)

**Figure 4:**
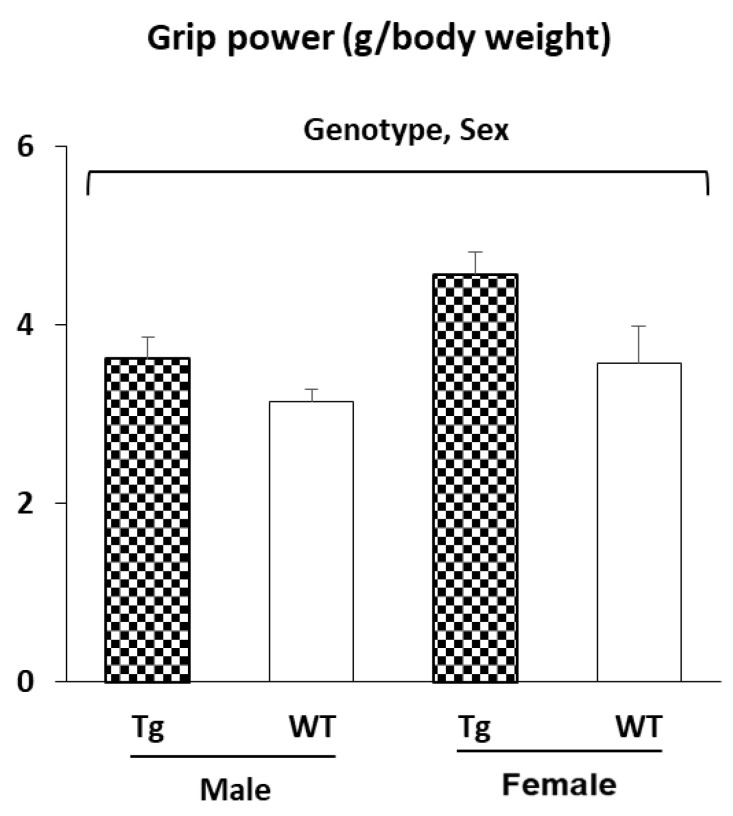
Muscle strength (grip power test): *Sparc* overexpression increased the grip power. Female mice had a higher grip power per body weight than male mice. All data are mean ± SEM. The number of mice: 5–11 mice per experimental condition. Abbreviations: g, gram; *Sparc*, secreted protein acidic and rich in cysteine; Tg, transgenic (*Sparc* overexpression); WT, wild-type.

The mice overexpressing *Sparc* had a greater grip power, and the female mice had a higher grip power than the male mice (Figure 4). The grip power is given as a muscle strength divided by the body weight.

#### 2.2.3. Glycemia, Glucose Transporter Type 4 (GLUT4), and Mitochondrially Encoded Cytochrome c Oxidase II (MT-CO2) Expression in the Muscle

We measured the fasting glycemia along with the protein expression of both the GLUT4 and the MT-CO2. These measures are a part of a set of parameters that—together with our designed context—support our conclusions.

**Figure 5:**
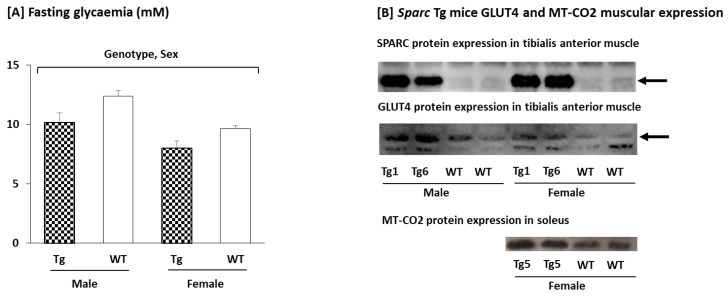
Glycemia as well as glucose transporter type 4 (GLUT4) and mitochondrially encoded cytochrome c oxidase II (MT-CO2) expressions in the muscle of F1 and F2 mice. As metabolic indicators, our measures showed that *Sparc* overexpression leads to a lower blood glucose (**A**) along with higher expressions of both GLUT4 and MT-CO2 (**B**). Glucose is lower in females compared to males as well (**A**). We also confirmed that SPARC is constantly overexpressed in *Sparc* Tg mice regardless of generation or sex (**B**). For the glycemia, all data are mean ± SEM, and the number of mice: 5–11 mice per experimental condition. Abbreviations: SPARC/*Sparc*, secreted protein acidic and rich in cysteine; Tg, transgenic (*Sparc* overexpression); WT, wild-type. Tg1: *Sparc* Tg line 1, Tg5: *Sparc* Tg line 5, Tg6: *Sparc* Tg line 6.

The *Sparc* overexpressed mice had a lower fasting blood glucose level along with higher expressions of both the GLUT4 and the MT-CO2 (Figure 5). The female mice had a lower fasting glucose level than the male mice. We also confirmed that SPARC is constantly overexpressed in the *Sparc* Tg mice, regardless of generation or sex. The SPARC expression also correlated with the GLUT4 and MT-CO2 expression, which confirms the genotype effect.

## 3. Discussion

For the *Sparc* Tg experiment, the male mice had a higher body weight than the female mice (Figure S1). This could have impacted the statistical analyses of IngAT and muscle (higher in males) (Figure 3) as well as the grip power (lower in males) as the grip power was divided by the body weight (Figure 4). Thus, the sex effect will not be discussed because we already have a sex effect on the body weight. We will focus, rather, on the genotype (*Sparc* overexpression) effect in the discussions. For the *Sparc* KO experiment, the discussions will include the three variables (genotype, age, and exercise).

The roles that SPARC plays at various levels, as shown by the published evidences, allowed us to predicate some of the impacts that SPARC deficiency and SPARC overexpression would have. These impacts are to be explored to build/optimize animal models of health conditions or diseases as well as to be starting points towards novel molecular therapies. Our data, compiled with the previous evidences, allow us to suggest new SPARC-related models and applications. As illustrated below, we highlight that *Sparc* KO mice would represent (accelerated) ageing animal models. After that, we also give other examples of how *Sparc* KO mice could be models, or a starting point to build models, of other diseases or health conditions as well. 

### 3.1. Sparc KO to Optimize (Accelerated) Ageing Animal Models

Ageing is the biological process of the progressive loss of homeostatic and metabolic functions, along with compromised physical performance and physiological integrity [40,41], such as a decline in aerobic capacity [3], reduced muscle oxidative capacity [2], and imbalanced glucose utilization [6]. It leads to new cellular, metabolic, and molecular (including transcriptional [42]) patterns, with a pathophysiological predisposition for diseases [43,44]. Therefore, understanding the underlying mechanisms of ageing and the related metabolic and structural properties is very important to optimize healthcare. In this context, various (accelerated) ageing animal models have been developed to study ageing [45], especially mouse models [46,47]. They represent animals that have been modified (such as with chemicals [48]) in order to biologically mimic what is seen during ageing.

On the other hand, our previous [11] and current in vivo results showed that ageing increased adiposity and body weight but decreased muscle mass, strength, glucose tolerance (in WT mice), and glucose muscular uptake (GLUT4). To highlight the similarities between ageing effects (either shown from our data or from the literature) and *Sparc* KO, our data, as well as the results we previously reported from the same set of *Sparc* KO experiment mice [11], showed that *Sparc* KO led to an ageing-like phenotype, including muscle loss, grip power reduction, lower oxidative phosphorylation, glucose intolerance, and reduced glucose transport into muscles. 

A part of the ageing-like phenotype is the similarity between the *Sparc* KO phenotype and sarcopenia. Sarcopenia, for which ageing represents a risk factor [37], is characterized by a generalized loss of muscle mass and strength [49]. This clinical definition fits with the *Sparc* KO phenotype (low muscle mass, reduced muscle metabolic performance, and reduced muscle strength) as we reported previously [11] on the signs that are also related to both ageing and a sedentary lifestyle [39], which are impacted by myokines, including SPARC [39]. The *Sparc* KO consequences on muscle are probably due, in part, to the interaction between SPARC and actin, which are important for muscle function [50]. Regarding the *Sparc* KO mice, we saw an increased glycogen content in the muscles in addition to the muscle loss. Thus, not only did the *Sparc* KO mice have lower muscle weight, but a part of that weight was glycogen, which suggests further reduced mechanical properties, as shown by the reduced muscle strength in *Sparc* KO mice [11]. Moreover, the impact of the SPARC deficiency could also contribute to the reduced grip-power performance of the *Sparc* KO mice. Indeed, the extracellular matrix collagen fibrils are important in tendons [51,52]. Thus, the interaction of SPARC with both the extracellular matrix and the collagen would contribute to the impact *Sparc* KO had on the Achilles tendon (reduced weight that could reflect a lack of development) and eventually even impact the grip power (also decreased in the *Sparc* KO mice [11]). In particular, SPARC is required for tendon mechanobiology, and its deficiency impairs tendon maturation [53].

The declined mitochondrial functions (MT-CO1 [11]) in the *Sparc* KO mice (considered as an ageing model), as well as the anti-SPARC antibody-induced decrease in the expression of mitochondrial proteins, ubiquinol-cytochrome c reductase core protein II (UQCRC2) and succinate dehydrogenase iron-sulfur subunit (SDHB) in muscle cells [13], support the mitochondrial theory of ageing [54] and further highlight SPARC as an important factor in the age-related changes, as illustrated by the decreased myogenesis and myogenin expression in the C2C12 muscle cells following the treatment with the anti-SPARC antibody [13]. The glucose intolerance (revealed by the OGTT) in *Sparc* KO mice also mimics ageing-related glucose intolerance [55].

For the adiposity, our data show that the *Sparc* KO reduced the AT weight and the adipocyte number only in the visceral AT (EpiAT) and not the subcutaneous AT (IngAT), a pattern that can be explained by the suggestion that SPARC expression in the AT is predominant in the subcutaneous AT [56], as shown by the SPARC mRNA expression that was significantly higher in the subcutaneous abdominal adipose tissue compared to the visceral adipose tissue [57]. The property SPARC had on white AT, and also on BAT, towards improving their metabolic performance would also have consequences. Indeed, whereas SPARC induced lipolysis, fat oxidation, and browning in the white adipocytes and BAT activation, its knockdown reduced the markers of these metabolic pathways [58]. Thus, *Sparc* KO would be towards adiposity development resulting from a poor local metabolic rate of the adipose tissues. In our *Sparc* KO mice, we have an increased BAT weight percentage that would rather be a white AT depot inside the BAT areas (infiltration) that could reflect a *Sparc* KO-dependent fat distribution. For the BAT, we still have the effect of the exercise that increased (percentage weight). Together (BAT increase with exercise and decrease with *Sparc* KO), these elements support the fact that our BAT was infiltrated with white AT (two tissues together in the BAT location) and that the exercise-induced increase is due to BAT, whereas the *Sparc* KO-induced increase is due to the infiltrating of white AT into BAT location. This is confirmed by the interaction between the genotype and exercise effects that reveal that the *Sparc* KO increased BAT only in the exercised mice and not in the sedentary mice. On the other hand, although ageing tends towards body fat increase [59], the *Sparc* KO reduced visceral adiposity (AbdAT). This can be explained by the fact that the *Sparc* KO mice have a higher glycogen content, suggesting that the energy storage takes the form of muscular glycogen (suggested explanation in Section 3.2) rather than fat storage in adipocytes. Although AT is heterogenic in male C57BL/6J mice (that we have used) [60] and its growth has a dynamic between hypertrophy and hyperplasia [61], we have seen no genotype effect on the EpiAT and IngAT adipocytes size. This supports our hypothesis, which states that to see a SPARC deficiency-related difference in the adipocyte, we need to induce obesity as HFD-induced obesity is enhanced in the absence of SPARC [62]. The high muscle mass (storage ability) percentage (in humans, it is 40% of total body weight [37]) compared to the body fat also supports this energy storage redistribution from adiposity to muscle glycogen seen in *Sparc* KO, especially given that our mice were fed a chow diet and not a high-fat diet (HFD). Indeed, HFD-induced obesity is enhanced in the absence of SPARC [62]. Therefore, non-similarity between the age-related changes and the *Sparc* KO impacts of adiposity in our study can be overcome by feeding mice HFD and building a new age-related animal model of sarcopenic obesity (Section 3.3).

Moreover, SPARC declines with ageing would also be implicated in the age-related decline in muscle cell regeneration [63,64] as SPARC has been pointed out as a regeneration factor [65] and, whereas the anti-SPARC antibody prevented the differentiation of C2C12 myoblasts, adding SPARC increased the differentiation [13]. Importantly, for the properties in which we had an interaction between age and genotype, the *Sparc* KO effects are more significant in the young mice compared to the old mice. This is explained by the fact that SPARC declines with ageing and, thus, SPARC deficiency will have more impacts in young mice compared to old/aged mice that already have an age-related reduced SPARC expression. This is seen, for instance, in AbdAT, RetAT, EpiAT, and MesAT, where the difference in AT weight between old and young is more important in the WT mice (KO reduces the age-dependent difference). 

Moreover, other data of the literature report other ageing-like changes that result from SPARC deficiency, such as reduced bone formation and osteoblast number [16], osteopenia [25,66], diminished levels of collagen [62], early onset of cataracts [21,67], immune alterations [68], and accelerated degeneration [69,70]. Thus, this further highlights the similarities between ageing biological patterns and SPARC deficiency and supports the classification of *Sparc* KO mice as an accelerated ageing animal model. 

### 3.2. Sparc KO as Type 1 Diabetes Model?

As impaired glucose tolerance has been reported in SPARC-deficient mice [62,71] and Atorrasagasti et al. showed evidence that SPARC deficiency produced diabetic mice [71]. However, they also showed that the mice had an impaired insulin-secretion capacity rather than insulin resistance (on HFD) [71]. Such findings point to a type 1 diabetes-like phenotype (rather than type 2 diabetes), especially as it has been suggested that SPARC plays a role in insulin secretion [72] and promotes insulin secretion in pancreatic β cells [73]. Therefore, SPARC deficiency would reduce insulin secretion (independently of insulin resistance) [72].

As glucose intolerance characterises diabetes [74] and OGTT is one of the most widely used methods to characterise diabetes mouse models [75], we performed an OGTT, along with other metabolic measures, during our *Sparc* KO study. Our data support the findings of Atorrasagasti et al. Indeed, we have found that *Sparc* KO reduced both glucose tolerance as well as glucose uptake (GLUT4). The analysis of the interaction between the age, genotype, and exercise shows that there is no difference between the young and old mice of the *Sparc* KO sedentary mice, but in the *Sparc* KO exercise mice, the old mice had a better glucose tolerance than the young mice. This is explained by the higher expression of SPARC in the young mice compared to the old mice. Thus, SPARC deficiency would have a greater impact on the younger mice. The exploration of the mitochondrial metabolism revealed that *Sparc* KO reduced the expression of MT-COI (mitochiondrial oxidative phosphorylation) [11] and also increased glycogen storage in muscle. This *Sparc* KO-induced increase in the glycogen content in the muscle suggests that, overall, *Sparc* KO shifts the metabolic homeostasis from glucose utilization/AT fat storage towards muscle glycogen storage. It might indicate that SPARC is required for the glycogen breakdown and/or that SPARC deficiency would increase glycogenesis and/or reduce glycogenolysis. The *Sparc* KO-induced increase in muscle glycogen requires further investigation, which could lead to the elucidation of metabolic patterns that would further add to the mechanistic explanations of how SPARC deficiency impacts glucose metabolism (decreased glucose tolerance, mitochondrial metabolism [11], and glucose GLUT4 uptake) and lead to both abnormal glucose metabolism [71] and glycogen usage, especially after researchers started investigating the links between SPARC and skeletal muscle glycogen breakdown [76]. It is worth noting that the increased glucose intolerance in our *Sparc* KO mice correlates with the decreased GLUT4 expression in the same mice and the previously reported impaired insulin secretion in SPARC-deficient mice [71]. The declined metabolic performance of the muscle, combined with the diabetes-like phenotype in the *Sparc* KO mice, would limit their need for glycogenolysis-released glucose and, thus, might conserve the glycogen storage more than in the WT mice. This could also explain the increased glycogen content in the muscles. In addition, *Sparc* KO amplified the HFD-induced obesity [62]. Therefore, a combination of *Sparc* KO and a HFD could further optimize such an impaired-glucose homeostasis model (add insulin resistance), knowing the links between obesity and diabetes through either HFD-induced or obesity-related insulin resistance. 

### 3.3. Sparc KO to Optimize Sarcopenic Obesity 

Sarcopenic obesity is an age-related health problem in which we see a combination of obesity with a reduced muscle mass and strength [77]. Above, we have detailed similarities between the *Sparc* KO muscle phenotype and sarcopenia. The enhanced HFD-induced obesity in *Sparc* KO mice [62] would be a good example of how *Sparc* KO mice can be a starting model from which to build an optimized model on it. It indicates that we can apply the diverse method used to generate obesity models [78] on the *Sparc* KO mice to create an obesity model with enhanced obesity features. The model of the *Sparc* KO will manifest reduced muscle mass and muscle power [11] combined with the enhancement of HFD-induced obesity [62]. Therefore, combining the KO (and even a knockdown) of *Sparc* and an HFD would build a sarcopenic obesity animal model. In addition, such models would be further optimized in aged animals (compared to young animals), especially given that ageing and obesity have shared patterns [79]. The advantages of such an animal model are that it would have the two features of the disease that are enhanced. Indeed, whereas the ageing would be accelerated with the *Sparc* KO, the HFD-induced obesity will also be enhanced by the *Sparc* KO. This is of particular importance because we can create two conditions with one approach, *Sparc* KO (instead of using one method to generate sarcopenia and another one to enhance obesity), which will reduce the variabilities and the limitations of such an animal model.

### 3.4. SPARC Overexpression Mimics Exercise Benefits

In our *Sparc* Tg mice that overexpress SPARC, we did not observe any side effects of the *Sparc* Tg, even up to 10 months old. In addition, for the body weight as well as the key metabolic and functional tissues, including the heart, liver, kidney, pancreas, spleen, and femur, there is no impact of the SPARC overexpression on tissue weights (data not shown), which would reflect no abnormal growth and the safety of overexpressing SPARC in terms of possible carcinogenesis. In fact, we have previously reviewed data that even suggested an antitumor character of SPARC [80].

It has already been suggested that SPARC treatment mimics the exercise effects by enhancing the glucose uptake (and glucose tolerance) in skeletal muscle [81]. This correlates with the increase in GLUT4 expression and the lower glycemia seen in our *Sparc* Tg mice. It also fits with the exercise-induced GLUT4-increased expression [82] and the SPARC-induced increase in glucose uptake and GLUT4 levels combined with the SPARC knockdown-induced GLUT4 protein level reduction [30]. The overexpression of SPARC in our Tg mice also leads to an increased muscle weight (trend), muscle strength, and mitochondrial oxidative phosphorylation (MT-CO2) expression, along with reduced IngAT in male mice (trend). Moreover, our previous in vitro study showed that adding or inducing SPARC in C2C12 muscle cells increased their differentiation, myogenin expression, collagen expression, and the expression of two mitochondrial proteins (UQCRC2 and SDHB) [13] and a mitochondrial biogenesis master regulator (PGC-1α) [8]. The reduced adiposity in Tg mice correlated with the property SPARC had on white AT and also on BAT towards improving their metabolic performance (lipolysis, fat oxidation, and browning in white adipocytes and BAT activation [58]). Similarly, our previous [11] and current in vivo results indicated that whereas exercise improved muscle (TA) mass, grip power (trend), glucose tolerance, collagen expression (trend), glucose uptake (GLUT4 expression), and mitochondrial oxidative phosphorylation (MT-CO1 expression), it reduced adiposity and body weight (trend). In addition, it has already been documented that exercise increased muscle mass [83], muscle oxidative capacity [2], muscle strength [84], and mitochondrial oxidative phosphorylation [11]. Exercise also reduced adiposity [85] and increased muscle differentiation [86], mitochondrial biogenesis [87] and, in general, attenuated age-related decreases in muscle properties (regeneration, metabolism, strength, and mass) [41]. All together, these properties show key similarities between SPARC overexpression and exercise benefits and exemplify how exercise-induced myokines (including SPARC) can explain the impacts of exercise, especially in the aged population [88] and on metabolism [89]. In addition, SPARC has been pointed to in the mediation of other effects that are also produced by exercise, such as tumor growth suppression [80,90] and the anti-inflammatory effect [91].

Such observations have two main implications. First, they further support the implication of SPARC as a key biomolecular mediator of the exercise benefits and place SPARC as the mechanistic link between exercise and its benefits. This leads us to the second implication, which is the possible therapeutic use of SPARC or its related pathways to induce exercise-like effects without performing exercise. The benefits would also include other exercise-related benefits such as tumor growth suppression [90]. 

## 4. Materials and Methods

Our work is divided into two parts. First, we explore the effects of *Sparc* knockout (KO) and compare them to the ageing consequences. We also observe the effects of exercise. In the second part, we study the effects of *Sparc* overexpression and compare them to the exercise benefits. At the end, we make combinatory analyses of the results to point out the analogies between the *Sparc* KO and the ageing-like phenotype on the one hand and make comparisons between *Sparc* overexpression and exercise in the context of exercise counteracting ageing.

**Figure 6:**
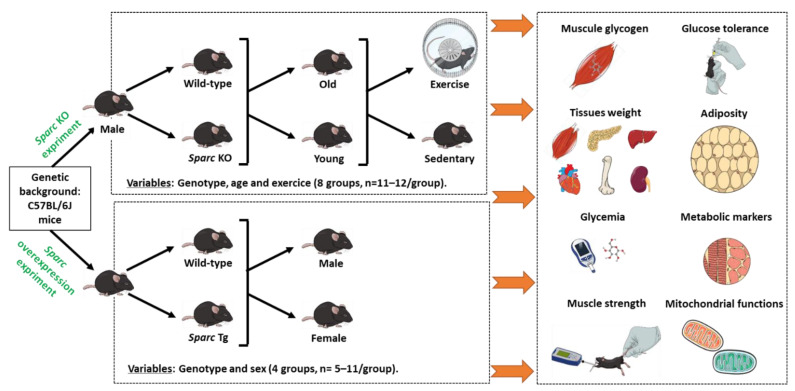
Experimental designs to explore the impacts of *Sparc* KO (in the context of ageing and exercise) as well as the impacts of *Sparc* overexpression in both male and female mice. For each experiment, a number of parameters have been conducted. Abbreviations: KO, knockout; *Sparc*, secreted protein acidic and rich in cysteine; Tg, transgenic (*Sparc* overexpression).

To optimize the results of both parts towards the combinatory conclusions, the mice of both studies had the same genetic background, C57BL/6J. The C57BL/6J mouse, with a lifespan of around 104 weeks (26 months) [92,93], is the most commonly used strain for genetic and/or transgenic study that also consistently shows the highest level of voluntary wheel-running (suitable for exercise) [94], and in this strain, exercise capacity declines with age [95]. These properties make C57BL/6J mouse strain suitable for our studies, which both use transgenic mice and explore exercise effects. Furthermore, the mice of both studies were housed at the same animal facility of the CHU de Québec-Université Laval Research Center (12 h light/dark cycle), under the same conditions and fed with the same chow diet (Teklad global 18% protein rodent diets [96]) and had access to food and water ad libitum during the whole experimental period (except for fasting periods, during which they had access to water only). All the mice were sacrificed by cardiac puncture following isoflurane inhalation anesthesia. Figure 6 summarizes the key experimental designs that we detail below. 

### 4.1. Sparc KO Experiment

The first study was carried out on biological samples from the mice used in our previous study. The previous study we conducted mainly focused on the effects of exercise patterns and suggested that exercise-induced muscle phenotype changes are SPARC-dependent [11]. In this continuation, we aim to rather identify the potential similarities between the *Sparc KO* phenotype and ageing. Therefore, we performed additional measures towards a set of data to validate our hypothesis linking *Sparc* KO to an ageing-like phenotype. Briefly, the study was carried out on male mice and involved both WT mice (C57BL/6J) and *Sparc* KO mice (129/Sv-C57BL/6J). Whereas the WT mice were from the Jackson Laboratory (https://www.jax.org/, Accessed date: 31 December 2021), the *Sparc* KO mice were generated via in vitro fertilization using *Sparc* KO mice sperm generously provided by Dr. Amy D. Bradshaw, who generated the *Sparc* KO mice, as previously described [62,97]. Each age-group of mice (young (Y) and old (O)) was divided based on the genotype (KO or WT) to obtain 4 groups: Y-KO, Y-WT, O-KO, and O-WT. Finally, each of these 4 groups was further subdivided into two groups according to whether they were exercising (Ex) or sedentary (Sed) mice. Therefore, our experimental design included 8 groups: Y-WT-Sed, Y-WT-Ex, Y-KO-Sed, Y-KO-Ex, O-WT-Sed, O-WT-Ex, O-KO-Sed, and O-KO-Ex. Each group had 11 to 12 mice (Figure 6).

The exercising mice were trained (starting at the age of 9 weeks for the young mice and 66 weeks for the old mice) on running wheels (Lafayette instrument Co, Lafayette, IN, USA) placed horizontally. The exercise groups were trained during the dark phase. The exercising mice were trained for a total of 12 weeks. Based on previously reported protocols, also applied to C57BL/6J mice [94,95], we first determined the speed at the lactate threshold (LT), at which the mice were trained afterwards on running wheels. The training speed was fixed as the LT intensity based on the benefits and effects that such an exercise pattern has been shown to produce, including those on glucose effectiveness, body fat percentage, insulin sensitivity, blood pressure, physical fitness, and lipid profile [7,98,99,100,101]. The LT level was also the speed used during the study during which *SPARC* has been characterised as an exercise-induced gene [7]. Moreover, as this part mainly focuses on exploring the potential similarities between the *Sparc* KO phenotype and ageing, the LT speed was selected for the exercise as its intensity can be prescribed for an older person [99].

To optimize our design, the sedentary mice were also taken to the training room to experiment with similar stimuli (auditory, olfactory, photonic, etc.) to that of the trained mice during the exercise sessions (60 min/day, five times/week). The running was mainly voluntary as no potentially harmful stimulations (such as electricicty [95]) were applied to force the mice to run. To ensure that all the mice ran at the same speed and during the same period (similar exercise amounts), we used a small hand air pump to apply a light air stimulation a few times during the training sessions. We chose to conduct this part of our investigation on male mice because we aimed to explore the muscles in the context of exercise and ageing. Skeletal muscle development and response to exercise, as well as sarcopenic (age-related) muscle mass and strength loss, are more important in males compared to females [102,103]. In addition, exercise-induced SPARC levels correlate with skeletal muscle mass [104]. Therefore, a decline (*Sparc* KO/ageing) or enhancement (exercise) in functions and metabolic performances of the muscle would be more likely to be seen in males. 

#### 4.1.1. Mice Sacrifice Tissue Weights

The mice were sacrificed at the age of 21 weeks for the young mice and at the age of 78 weeks for the old mice. They were sacrificed 48 h after the end of the 12 weeks of exercise training to avoid the acute impacts of the exercise on the measurements conducted afterwards. Immediately after the sacrifice of each mouse, its tissues were weighed. The tissues of concern were the Achilles tendon, brown adipose tissue (BAT), inguinal adipose tissue (IngAT), abdominal adipose tissue (AbdAT), retroperitoneal adipose tissue (RetAT), epididymal adipose tissue (Epi AT), and Mesenteric adipose tissue (MesAT) and the three muscles, gastrocnemius (GC), soleus (Sol), and extensor digitorum longus (EDL). As for the tissues that were used later for either Western blot or glycogen quantification, they were snap frozen in liquid nitrogen then moved to −80 °C and stored until use.

#### 4.1.2. Oral Glucose Tolerance Test (OGTT)

In order to evaluate the glucose tolerance in mice [75], we performed an oral glucose tolerance test (OGTT). The mice were fasted for 6 h prior to the glucose gavage. After the glucose (prepared from 45% solution, Sigma-Aldrich Canada Co., Oakville, ON, Canada) oral gavage (2 mg of glucose per 1 g of body weight), we measured the glycemia 5 times: 0, 15, 30, 60, and 120 min after glucose gavage. These 5 timepoints allowed us to obtain a curve and calculate the area under the curve (AUC) [105]. The OGTTs were administered three times (a total of three AUCs), at pre-training, at week 5, and at the end of week 12 of the training. The blood glucose levels were measured via tail pricking with a needle to collect blood samples on glucose test strips that were then inserted into a blood glucose meter (Accu-Chek, Roche Diabetes Care, Inc., Mississauga, ON, Canada) to read the blood glucose levels.

#### 4.1.3. Muscle Glucose Transporter Type 4 (GLUT4) Expression (Western Blot) 

The glucose transporter type 4 (GLUT4) is a transporter that increases with exercise training, allowing glucose to enter the muscles [82]. We measured the expression of GLUT4 in the tibialis anterior (TA) muscle because it is the skeletal muscle for which we previously had an effect of age (decrease), genotype (decrease), and exercise (increase) on either its weight or its weight percentage [11]. To measure the expression of GLUT4, the total proteins were extracted from the TA muscle, using a radio-immunoprecipitation assay (RIPA) buffer and a protease inhibitor cocktail (Sigma-Aldrich Canada Co.), and followed by a protein quantification of each protein extract using a Bio-Rad protein assay (Bio-Rad Laboratories Ltd., Mississauga, ON, Canada). The protein extracts were kept at −80 °C until the Western blot was performed. Five micrograms of proteins was separated by sodium dodecyl sulfate polyacrylamide gel electrophoresis (SDS-PAGE) using the TGX Stain-Free FastCast acrylamide solutions (Bio-Rad Laboratories Ltd.), and the trihalo compound in the gels was activated under UV light. Then, the total proteins were transferred to polyvinylidene fluoride (PVDF) membranes (Bio-Rad Laboratories Ltd.), and the gels (before and after the transfer) and membranes were visualized under UV light by using the AlphaImager TM 1220 (Alpha Innotech Co., San Leandro, CA, USA). The membranes were blocked using the Pierce™ Protein-Free (TBS) blocking buffer (Life Technologies Inc., Burlington, ON, Canada), incubated overnight with 1/1600 dilution (in the blocking buffer) of the primary antibody (sc-7938, Santa Cruz Biotechnology Inc., Dallas, TX, USA) and a 1 h incubation with a 1/10,000 dilution (in the blocking buffer) of the secondary antibody (sc-2004, Santa Cruz Biotechnology Inc.) and finally visualized with the Clarity™ Western ECL Blotting Substrate on a film (Bio-Rad Laboratories Ltd.). As the total number of mice (samples) was 95, we used many gels to load all the samples. Therefore, we needed an intermembranes and interfilms normalization method to both optimize and quantify these Western blot results. First, the visualized total proteins on the membranes and target proteins on the films were quantified using ImageJ software (ImageJ bundled with 64-bit Java 1.8.0_172, U. S. National Institutes of Health, Bethesda, MD, USA) [106]. The methodology of the lane and band quantifications, followed by the expression evaluations, was performed according to Taylor et al. [107,108], as we have detailed in one of our previous works [109].

#### 4.1.4. Skeletal Muscle Glycogen Content

In order to further explore the metabolic phenotype of the muscle, we measured the glycogen content in the gastrocnemius (GC) muscle. The method was based on the protocol previously published [110]. We used the standard curve to quantify the glycogen in the solution. We divided the glycogen content by the GC weight to have the glycogen content in μg per mg of muscle.

#### 4.1.5. Histological Analysis of the White Adipose Tissue

We analyzed both the inguinal and the epididymal adipocytes. These two locations are representative of the subcutaneous and visceral adipocytes, respectively. Immediately after the sacrifice, the adipose tissue depots (inguinal and epididymal) were harvested and fixed in 4% paraformaldehyde for 48 h and then paraffin-embedded for use in histology (hematoxylin and eosin staining). Slices of 5 µm were prepared for the paraffin-embedded tissue sections. The section images were taken using the microscope Nikon Eclipse E800 (magnification of ×20) combined with a digital camera Nikon D5500, Nikon corporation (magnification of ×2). The cross-sectional area analysis of the adipocyte diamaters was performed using the ImageJ [106]. For each mouse, we had 5 images, and we analysed the cross-sectional area of 7 adipocytes from each image. In total, we had 35 cross-sectional areas for each adipose tissue sample. We also, based on the cross-sectional area and the adipose tissue weight of each mouse, estimated the number of the adipocytes [111]. 

#### 4.1.6. Statistical Analyses

The data were analyzed by three-way (age, genotype, and exercise) and four-way (plus time for the OGTT) ANOVA. When the ANOVA revealed a significant interaction between two or three variables, the Tukey Kramer post hoc test was performed to identify the significant difference between the groups (*p* < 0.05). A trend corresponds to 0.05 ≤ *p* < 0.1. In the results section, all the effects are significant (*p* < 0.05), unless mentioned as a trend. The number of mice (11–12 mice per experimental condition) was based on the results of a power analysis by setting the statistical power at 80% (α = 0.05 and β = 0.2) with our previous study, which used the same strain of WT mice [112].

### 4.2. Sparc Overexpression (Sparc Tg) Experiment

We used transgenic mice overexpressing *Sparc* (*Sparc* Tg) and WT mice, both with the same genetic background as those used in the *Sparc* KO experiment. The PiggyBac transposase-mediated gene transfer was used to create transgenic lines expressing mouse *Sparc* under the control of a strong and ubiquitous CAG promoter (CMV early enhancer fused to modified chicken b-actin promoter) in a C57BL/6 mouse. In the PiggyBac vector, the “CAG promoter-Kozak-mouse *Sparc* CDS-polyA” cassette was flanked by two PiggyBac inverted terminal repeat sequences (ITRs) to facilitate the transpose-mediated transgene integration. The verified PiggyBac vector carrying the transgenic cassette was co-injected with transposases into the pronucleus of fertilized eggs, and the eggs were implanted into surrogate mothers to obtain offspring. The pups were genotyped by PCR to identify the ones carrying the desired PiggyBac transgene. The positive founder mice were counter screened for transposes. Out of 25 pups screened, 6 were identified positive (Tg1 to Tg6, 4 males, and 2 females). The procedure above was performed by Cyagen Biosciences [113].

#### 4.2.1. Confirming the SPARC/*Sparc* Gene Overexpression and Selecting Mice (Western blot and Q_RT-PCR) 

After all 4 male and 2 female *Sparc* Tg mice were transfered to our animal facility, the F1 mice were produced with an average of 8.4 F1 pups per F0 mouse (the ratio of male to female pups was 0.84). As no known toxic component was included in this design, the only risk was that the transgenic cassette could have been randomly inserted into the genome and may have disrupted the endogenous genes. This is why we used multiple transgenic founders to minimize this artificial effect. The overall expression level in various tissues will be affected by the regulatory mechanism of the CAG promoters and the turnover time of the protein itself, which could be variable in every tissue. Thus, we also investigated the SPARC expression in several tissues using quantitative real-time PCR (Q_RT-PCR) and/or Western blot.

For the Western blot, we measured the expression of SPARC in the TA, heart, liver, BAT, IngAT, and gonadal (Gon) AT. On the day of the sacrifice, the tissues were removed and quickly put in liquid nitrogen (snap frozen), then moved to −80 °C and kept until the protein extraction procedure (See Section 4.1.3). The protein extracts were kept at −80 °C until the Western blot was performed. First, 20 and 40 micrograms of TA proteins (extract) of 7 mice (Tg1, Tg2, Tg4, Tg5, Tg6, and two WT) were separated by SDS-PAGE using the TGX Stain-Free FastCast acrylamide solutions, and the trihalo compound in the gels was activated under UV light. Then, the total proteins were transferred to PVDF membranes, and gels (before and after the transfer), and the membranes were visualized under UV light. The membranes were blocked for 2 h in 2% nonfat dry milk (Bio-Rad Laboratories Ltd.), incubated overnight with 1/250 dilution (in Pierce^TM^ Protein-Free (TBS) blocking buffer) of the primary antibody (AF942, R&D Systems, Inc., Minneapolis, MN, USA), and a 2 h incubation with 1/1000 dilution (in Pierce^TM^ Protein-Free (TBS) blocking buffer) of the secondary antibody (sc-2354, Santa Cruz Biotechnology Inc.), and finally visualized with the Clarity™ Western ECL Blotting Substrate on a film. Unlike the first part of this paper (*Sparc* KO experiment), the number of samples for each target protein allowed us to have all the samples loaded on the same gel. Therefore, there was no need for a normalization. We confirmed the similarity of the loaded proteins by visualizing the protein lanes on the membrane after the transfer. The visualized target proteins on the films were quantified using ImageJ software [106].

For the *Sparc* gene expression analyzed by Q_RT-PCR, the tissues (TA) were homogenized in Qiazol buffer (Qiagen Inc., Germantown, MD, USA), and the total RNA was extracted using the miRNeasy micro kit on-column DNase (Qiagen Inc.) treatment following the manufacturer’s instructions. The quantity of total RNA was measured using a NanoDrop ND-1000 Spectrophotometer (NanoDrop Technologies, Wilmington, DE, USA) and total RNA quality was assayed on an Agilent BioAnalyzer 2100 (Agilent Technologies, Santa Clara, CA, USA). First-strand cDNA synthesis was accomplished using 4 ug of isolated RNA in a reaction containing 200 U of Superscript IV Rnase H-RT (Life Technologies), 300 ng of oligo-dT_18_, 50 ng of random hexamers, 50 mM Tris-HCl pH 8.3, 75 mM KCl, 3 mM MgCl_2_, 500 uM deoxynucleotides triphosphate, 5 mM dithiothreitol, and 40 U of Protector RNase inhibitor (Roche Diagnostics, Indianapolis, IN, USA) in a final volume of 50 uL. The reaction was incubated at 25 °C for 10 min, then at 50 °C for 20 min and inactivated at 80 °C for 10 min. A PCR purification kit (Qiagen Inc.) was used to purify the cDNA. The oligo primer pair that allows the amplification of 95 bp was designed by GeneTools software (Biotools, Edmonton, Alberta, Canada), and their specificity was verified by blast in the GenBank database. The gene name, GenBank accession number, and the sequences of the primer pair were the following: *Mus musculus* secreted acidic cysteine rich glycoprotein (*Sparc*), NM_009242, and CCACACGTTTCTTTGGACC/GATGTCCTGCTCCTTGATGC. The oligo primer pair was performed by IDT (Integrated DNA Technology, Coralville, IA, USA). A quantity corresponding to 20 ng of total RNA was used to perform fluorescent-based real-time PCR quantification using the LightCycler 480 (Roche Diagnostics). Reagent LightCycler 480 SYBRGreen I Master (Roche Diagnostics) was used as described by the manufacturer with 2% DMSO. The conditions for the PCR reactions were: 45 cycles, denaturation at 98 °C for 10 s, annealing at 57 °C for 10 s, and elongation at 72 °C for 14 s and then 74 °C for 5 s (reading). A melting curve was performed to assess the non-specific signal. The calculation of the number of copies of each mRNA was performed according to Luu-The et al. [114] using a second derivative method and a standard curve of Cp versus logarithm of the quantity. The standard curve is established using known amounts of purified PCR products (10, 10^2^, 10^3^, 10^4^, 10^5^, and 10^6^ copies) and a LightCycler 480 v1.5 program provided by the manufacturer (Roche Diagnostics). The PCR amplification efficiency was verified. The Q_RT-PCR measurements were performed by the CHU de Québec Research Center (CHUL) Gene Expression Platform, Quebec, Canada and were compliant with the MIQE guidelines [115,116]. 

**Figure 7:**
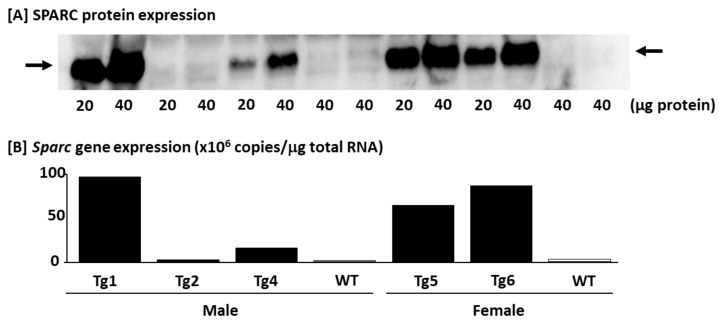
SPARC expression in the tibialis anterior muscle of *Sparc* transgenic (Tg) and wild-type (WT) F0 mice. (**A**) SPARC protein expression analyzed by Western blot. (**B**) *Sparc* gene expression analyzed by quantitative real-time PCR. Abbreviations: SPARC/*Sparc*, secreted protein acidic and rich in cysteine; Tg, transgenic (*Sparc* overexpression); Tg1, *Sparc* Tg line 1; Tg2, *Sparc* Tg line 2; Tg4, *Sparc* Tg line 4; Tg5, *Sparc* Tg line 5; Tg6, *Sparc* Tg line 6.

The results confirming the expression of SPARC and *Sparc* in the Tg lines are shown in Figure 7. As shown in Figure 7, we generated Tg mice expressing the *Sparc* gene at the different levels (*Sparc* Tg lines 1, 2, 4, 5, and 6 expressed the *Sparc* gene 69-, 1-, 7-, 31- and 39-fold compared to the WT, respectively), whereas the germline transmission did not occur in the *Sparc* Tg line 3. Therefore, *Sparc* Tg lines 2 and 3 were excluded.

**Figure 8:**
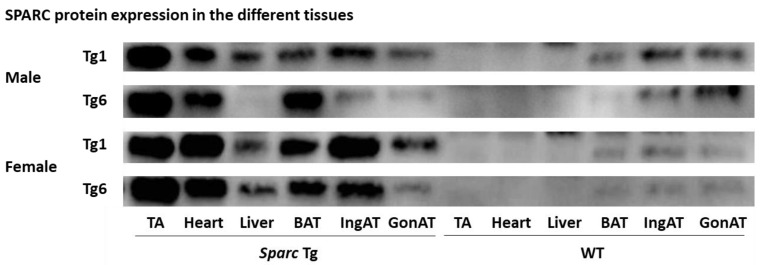
SPARC protein expression in the different tissues of transgenic (*Sparc* overexpression) and WT of F1 and F2 mice. Abbreviations: BAT, brown adipose tissue; GonAT, gonadal adipose tissue; IngAT, inguinal adipose tissue; TA, tibialis anterior; Tg, transgenic (*Sparc* overexpression); SPARC, secreted protein acidic and rich in cysteine; WT, wild-type.

In addition, to have an overview/confirm the SPARC expression in different tissues, we measured the expression of SPARC in six tissues (TA, heart, liver, BAT, IngAT, and GonAT) in 4 male and 4 female mice, 2 WT mice, 1 mouse from the line Tg1, and 1 mouse from the line Tg6. For WT mice sample run in parallel with Tg mice sample, both the WT and the Tg mice had a parent from a similar Tg line (Tg1 or Tg6), as shown in Figure 8. The lines Tg1 and Tg6 have been selected because they represent the lines that express the most SPARC/*Sparc* (Figure 7). The Western blot protocol of Figure 8 was the same as above (Figure 7) but with 15 µg as the protein loading amount.

At the end, the selected mice (for breeding and to generate both Tg and WT mice) all belong to the Tg lines 1, 5, or 6, which are the lines with the highest expression of SPARC/*Sparc* (Figure 7). For our study, the 32 mice constituted 4 groups, depending on genotype and sex, groups of males (M) or females (F), and Tg or WT (Figure 6). Therefore, we had four groups: Tg-M (*n* = 9), WT-M (*n* = 7), Tg-F (*n* = 11) and WT-F (*n* = 5). These were the numbers of mice for all our measurements except for those of Section 4.2.5. The average sacrifice age of each group was 6.0 ± 0.1 months. We ensured that there was no statistical age difference between the males and the females or between the WT and *Sparc* Tg mice (the two genotypes have similar ages). Therefore, a genotype effect would be explained only by the *Sparc* overexpression rather than age difference. 

#### 4.2.2. Body Weight and Tissue Weights

Living, fasted (12 h) mice were weighed just prior to their sacrifice. Immediately after the sacrifice of each mouse, its tissues were quickly removed and some of them weighed. The tissues of concern were skeletal muscles (GC, Sol, TA and EDL), IngAT, BAT, heart, liver, kidney, pancreas, spleen, and the femur. As for the tissues used later for Western blot, they were snap frozen in liquid nitrogen then moved to −80 °C and stored until use.

#### 4.2.3. Grip Power Test

Prior to their sacrifice, the muscle strengths of all the mice were measured through performing a grip power test with a grip strength meter (Columbus Instruments International, Columbus, OH, USA). The grip strength was measured by allowing the mouse to grab (with two limbs) pull bar assemblies attached to the force transducer while the mouse was pulled horizontally by the tail away from the bars, similar to what has previously been described [117], especially to detect physiological changes in the skeletal muscle function of C57BL/6J mice [118]. The peak force applied by the mouse (g) was then shown on a digital display. This test was conducted five times (5 min apart) for each mouse, after which the mean forces in grams were calculated as being normalized to the body weight of the corresponding mouse.

#### 4.2.4. Blood Glucose (Glycemia)

The mice were sacrificed following a 12 h fasting. At the end of the 12-h fasting and prior to the sacrifice, the blood glucose levels were measured via tail pricking with a needle to collect blood samples on glucose test strips that were then inserted into a blood glucose meter (Accu-Chek, Roche Diabetes Care, Inc. Mississauga, ON, Canada) to read the blood glucose value.

#### 4.2.5. Muscular GLUT4 and Mitochondrially Encoded Cytochrome c Oxidase II (MT-CO2) Expression (Western Blot)

Whereas GLUT4 is a transporter allowing glucose to enter the muscle [82], mitochondrially encoded cytochrome c oxidase II (MT-CO2) is a mitochondrial protein encoded by a mitochondrial gene that can be considered as an indicator of mitochondrial oxidative phosphorylation [119,120,121,122,123]. In addition to the glycemia, and in order to map the muscular metabolic patterns, we measured the TA expression of GLUT4 in 4 males and 4 females (2 WT mice, 1 mouse from the line Tg1, and 1 mouse from the line Tg6 for each sex) and the Soleus expression of MT-CO2 in 4 females (2 WT mice and 2 mice from the line Tg5). For a WT mice sample loaded in parallel with a Tg mice sample, they had a parent from a similar Tg line (Tg1, Tg5, or Tg6). We chose to measure the GLUT4 and MT-CO2 expression in the same muscles (TA and Soleus) where GLUT4 (above) and MT-CO1 [11] have been measured in a *Sparc* KO study to justify a later comparison of the results in our context. At the day of sacrifice, the two muscles were removed and quickly put in liquid nitrogen (snap frozen) then moved to −80 °C and kept until the protein extraction procedure. The Western blot steps were similar to the SPARC overexpression measures with different conditions. For GLUT4, 15 µg of proteins was loaded. The membranes were blocked using the Pierce™ Protein-Free (TBS) blocking buffer, incubated overnight with a 1/400 dilution of the primary antibody (sc-7938) before an incubation for 1 h with the secondary antibody (sc-2004) at a dilution of 1/10,000. For MT-CO2, 60 µg of proteins was loaded. The membranes were blocked using the Pierce™ Protein-Free (TBS) blocking buffer, incubated for 2 h with a 1/1600 dilution of primary antibody (sc-514489) before an incubation for 2 h with the secondary antibody (sc-516102) at a dilution of 1/10,000. All the antibodies were from Santa Cruz Biotechnology Inc. and diluted in Pierce™ Protein-Free (TBS) blocking buffer. Similarly to the SPARC protein expression quantification, the number of samples for each target protein allowed us to have all the samples loaded on the same gel. Therefore, there was no need for a normalization (similar to the one conducted in the *Sparc* KO Western blot experiment). We confirmed that there was no difference in protein loading by visualizing (under UV light) the protein lanes on the membrane after the transfer (rather than using beta-actin, for instance, that might not be a reliable loading control [124]). The visualized bands of the films were quantified using ImageJ software [106]. To better see the correlation between the SPARC expression and both the GLUT4 and the MT-CO2, the three proteins were observed in parallel.

#### 4.2.6. Statistical Analyses

The data were analyzed by two-way (genotype and sex) ANOVA. When the ANOVA revealed a significant interaction between two variables, the Tukey Kramer post hoc test was performed to identify the significant difference between the groups (*p* < 0.05). A trend corresponds to 0.05 ≤ *p* ≤ 0.1. In the results section, all the effects are significant (*p* < 0.05), unless mentioned as a trend.

## 5. Conclusions and Perspectives

The importance of SPARC, the various roles it plays, and its wide distribution makes its deficiency affect more than one system, and therefore, it leads to a relatively systemically-distributed phenotype rather than limited organ-specific or a tissue-specific changes.

**Figure 9:**
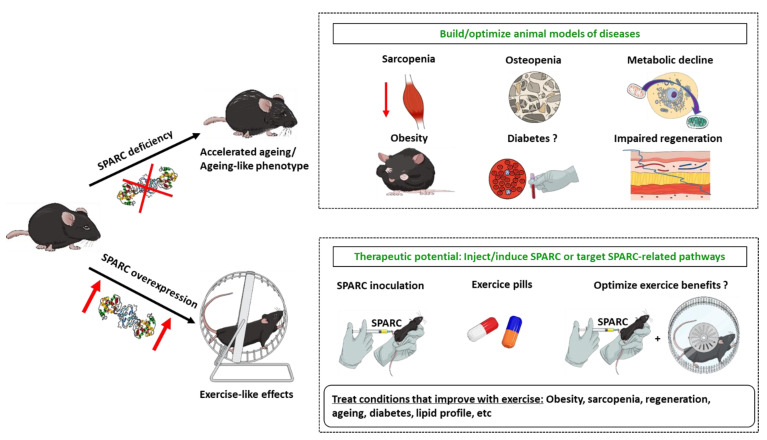
SPARC deficiency and ageing share similar phenotypes which are counteracted/improved by SPARC induction that mimics exercise effects. These can be exploited to both build/optimize animal models and potentially develop therapies to treat conditions that have common patterns with the SPARC-deficiency-related biological and functional changes. Abbreviations: SPARC, secreted protein acidic and rich in cysteine.

Our data highlight that SPARC is involved in both exercise-induced benefits, the ageing process, and the metabolic as well as the functional properties at various tissular and cellular levels. Indeed, SPARC declines with ageing and increases with exercise and its overexpression leads to an exercise-like effect. We noticed that *Sparc* KO and ageing lead to a similar phenotype in terms of reduced muscle mass, muscle power, and metabolic performance in addition to reduced glucose tolerance, glucose uptake, and collagen expression in the muscles. Furthermore, other in vivo and in vitro studies (reported above) on SPARC deficiency or inhibition highlight similar changes and have shown reduced muscle differentiation, decreased mitochondrial metabolism, osteopenia, and cataractogenesis, among other ageing-like patterns. On the other hand, SPARC overexpression in animals (this paper and our previous study [11]) or addition in cell culture (in vitro) [8,13] results in exercise-like effects, including enhanced mitochondrial oxidative phosphorylation, increased muscle mass and power, increased mitochondrial biogenesis, reduced glycemia and adipose tissue, increased glucose uptake by the muscle, and higher collagen and myogenin expression in muscle cells with increased myoblasts differentiation. All together, these evidences point out that *Sparc* KO leads to an ageing-like effect (or accelerated ageing) and that both the *Sparc* KO phenotype and ageing can be counteracted or reversed (at least in part) by either exercise or SPARC as SPARC-mediated effects mimic exercise. These can be exploited to both build/optimize animal models and potentially develop therapies (Figure 9). First, we could knockout or knock-down *Sparc* in order to build animal models (and even cellular models) to explore diseases and health conditions, as well as the pathways shown or suggested to interact with SPARC, such as its intracellular interactions and caspase-8, and in colon cancer cells [9], muscle AMPK signaling [81], integrin-linked kinase [8,26], RGS4 protein in pancreatic β cells [73], transforming growth factor-β1 in renal cell carcinoma [125], and beta-catenin in pulmonary fibroblasts [126]. This will deepen our understanding of the diseases and physiological conditions in which SPARC expression changes, such as cancer, obesity, muscle development, exercise, etc.

On the other hand, the exercise-like effects mediated by SPARC can open doors to use either SPARC or pharmacologically targeted SPARC-related pathways to achieve therapeutic goals that are usually obtained with physical exercise; in particular since SPARC has been suggested as a marker of exercise efficiency. Importantly, such benefits gave exercise the status of a therapeutic tool. The health problems and conditions for which exercise has been prescribed, and for which SPARC-related therapy can be explored to mimic exercise, include obesity, sarcopenia, diabetes, metabolic disorders, etc. This is of particular importance for those who need exercise but are unable to perform the prescribed amount because of physical disability, heart disease, or other health conditions. Indeed, such an “exercise pill” would overcome this challenge and still get the exercise benefits. Moreover, as SPARC would be required in the exercise-induced muscle changes, combining exercise with SPARC overexpression/inoculation could also improve and optimise exercise benefits. Importantly, the hypothesis we gave earlier to explain why SPARC is overexpressed in situations such as obesity [127] and cancer [80] is precisely an attempt to correct the damages induced by these situations through the beneficial properties of SPARC (metabolic enhancement, anti-cancer, etc.), as suggested by the increased plasma levels of SPARC in patients with newly diagnosed type 2 diabetes mellitus [128]. However, the non-identification of the SPARC receptor would represent a challenge that limits the exploration of SPARC, including using agonist or antagonist. Indeed, so far, putative receptors of SPARC have been reported [129,130], including alpha 5 beta 1 integrin complex (activates the Wnt/β-catenin), which has been identified as a candidate receptor [131]. Studies such as those overexpressing SPARC only in specific tissues and exploring the consequences of in vitro SPARC addition to cell/tissue cultures would also illuminate the path of understanding SPARC molecular patterns. 

It is worth noting that sequences of the *SPARC* gene and protein are highly conserved among species [22] and are aimed towards extrapolating from animal results to human studies. Importantly, with exercise considered as a panacea [132], SPARC seems to mediate and is likely to mimic exercise benefits. Elucidating SPARC pathways in the context of human diseases can add strong, yet safe, tools to the available therapeutic options for as many diseases and health problems as those for which exercise has been shown to have beneficial impacts. SPARC-based therapeutic pathways, with possible short comings, would be towards treating age-related health problems and metabolic disorders. Within this context, as SPARC expression was shown to have varying patterns based on targets and tissues, the rationale as to how to induce SPARC expression in selected tissues, or introduce it, can further optimize such a therapeutic approach. Indeed, based on SPARC-specific effects, including muscle growth, adipogenesis inhibition and anticancer, the development of new generations of pharmacological delivery systems would make a great contribution towards such novel molecular therapies by making them tissue-specific (muscles, tumors, etc.). That would increase the therapy precision, reduce possible side effects, and potentially lead to a personalised medicine.

## Figures and Tables

**Table 1:** Tissues weights and sizes.

		Young				Old				3-Way ANOVA				
		Wild-Type		*Sparc* Knockout		Wild-Type		*Sparc* Knockout						
		Sedentary	Exercise	Sedentary	Exercise	Sedentary	Exercise	Sedentary	Exercise	Age	Genotype	Exercise	Age × Genotype	Genotype × Exercise
Achilles tendon	mg	4.3 ± 0.6	4.0 ± 0.2	3.1 ± 0.2	2.8 ± 0.2	5.0 ± 0.2	5.0 ± 0.4	4.8 ± 0.2	5.1 ± 0.5	O ˃ Y	WT ˃ KO	-	WT: O > Y *, KO: O >> Y	-
	%	0.015 ± 0.002	0.014 ± 0.001	0.011 ± 0.001	0.011 ± 0.001	0.013 ± 0.001	0.014 ± 0.001	0.016 ± 0.001	0.017 ± 0.002	-	-	-	KO: O >> Y	-
Brown adipose tissue	mg	93 ± 13	103 ± 16	103 ± 8	130 ± 33	159 ± 27	142 ± 13	135 ± 12	192 ± 32	O ˃ Y	-	-	-	-
	%	0.31 ± 0.04	0.35 ± 0.05	0.37 ± 0.03	0.50 ± 0.10	0.41 ± 0.04	0.40 ± 0.03	0.42 ± 0.02	0.63 ± 0.10	O ˃ Y *	KO ˃ WT	Ex > Sed	-	Ex: KO > WT,
Inguinal adipose tissue	mg	19 ± 4	16 ± 4	18 ± 6	17 ± 3	41 ± 11	23 ± 1	22 ± 3	21 ± 2	O ˃ Y	-	-	-	-
	%	0.065 ± 0.014	0.054 ± 0.014	0.063 ± 0.023	0.068 ± 0.015	0.100 ± 0.020	0.065 ± 0.004	0.069 ± 0.008	0.069 ± 0.007	-	-	-	-	-
	µm^2^	1076 ± 109	1020 ± 126	1157 ± 87	1201 ± 102	3335 ± 646	3262 ± 542	2949 ± 613	1834 ± 215	O ˃ Y	-	-	WT: O > Y	-
	N	844 ± 140	471 ± 134	793 ± 206	411 ± 173	616 ± 144	332 ± 67	619 ± 161	578 ± 130	Y > O	-	-	-	-
Abdominal adipose tissue	mg	982 ± 204	781 ± 126	919 ± 133	651 ± 125	2831 ± 347	2603 ± 175	1760 ± 221	1463 ± 162	O ˃ Y	WT ˃ KO	Sed > Ex *	WT: O >> Y, KO: O > Y	-
	%	3.23 ± 0.58	2.61 ± 0.36	3.24 ± 0.43	2.49 ± 0.38	7.32 ± 0.63	7.31 ± 0.33	5.38 ± 0.49	4.71 ± 0.43	O ˃ Y	WT ˃ KO	-	WT: O >> Y, KO: O > Y	-
Retroperitoneal adipose tissue	mg	177 ± 38	125 ± 19	182 ± 34	119 ± 23	435 ± 40	410 ± 27	247 ± 30	230 ± 25	O ˃ Y	WT ˃ KO	Sed > Ex *	WT: O >> Y, KO: O > Y	-
	%	0.58 ± 0.11	0.42 ± 0.05	0.64 ± 0.11	0.46 ± 0.07	1.15 ± 0.08	1.15 ± 0.05	0.76 ± 0.07	0.74 ± 0.07	O ˃ Y	WT ˃ KO	-	WT: O >> Y, KO: O > Y	-
Epididymal adipose tissue	mg	305 ± 67	246 ± 42	293 ± 41	205 ± 39	870 ± 101	838 ± 54	556 ± 70	502 ± 58	O ˃ Y	WT ˃ KO	-	WT: O >> Y, KO: O > Y	-
	%	1.00 ± 0.19	0.82 ± 0.12	1.03 ± 0.13	0.78 ± 0.12	2.27 ± 0.21	2.36 ± 0.10	1.71 ± 0.17	1.61 ± 0.16	O ˃ Y	WT ˃ KO	-	WT: O >> Y, KO: O > Y	-
	µm^2^	2501 ± 196	2417 ± 226	3007 ± 230	2456 ± 255	4739 ± 635	4722 ± 512	4686 ± 562	4115 ± 287	O ˃ Y	-	-	-	-
	N	3397 ± 437	7037 ± 2648	2924 ± 291	6037 ± 2164	2553 ± 250	2856 ± 463	2392 ± 219	2805 ± 314	O > Y	WT > KO	-	WT: O > Y	-
Mesenteric adipose tissue	mg	195 ± 35	164 ± 25	152 ± 21	122 ± 25	656 ± 126	518 ± 46	400 ± 63	230 ± 31	O ˃ Y	WT ˃ KO	Sed > Ex	WT: O >> Y, KO: O > Y	-
	%	0.64 ± 0.10	0.55 ± 0.07	0.54 ± 0.07	0.46 ± 0.08	1.63 ± 0.22	1.44 ± 0.10	1.19 ± 0.14	0.74 ± 0.09	O ˃ Y	WT ˃ KO	Sed > Ex	WT: O >> Y, KO: O > Y	-
Gastrocnemius	mg	325 ± 9	316 ± 10	258 ± 8	244 ± 7	308 ± 7	307 ± 4	242 ± 5	237 ± 6	Y ˃ O	WT ˃ KO	-	-	-
	%	1.11 ± 0.04	1.10 ± 0.03	0.93 ± 0.04	0.99 ± 0.04	0.84 ± 0.04	0.88 ± 0.03	0.80 ± 0.04	0.78 ± 0.02	Y ˃ O	WT ˃ KO	-	-	-
Soleus	mg	11.5 ± 1.8	9.9 ± 0.2	9.1 ± 0.2	9.0 ± 0.4	10.7 ± 0.3	10.7 ± 0.3	9.0 ± 0.6	10.0 ± 0.6	-	WT ˃ KO	-	-	-
	%	0.039 ± 0.005	0.034 ± 0.001	0.033 ± 0.001	0.036 ± 0.001	0.029 ± 0.001	0.031 ± 0.001	0.029 ± 0.001	0.033 ± 0.002	Y ˃ O	-	-	-	Sed: WT > KO, Ex: KO > WT
Extensor digitorum longus	mg	25 ± 2	25 ± 2	20 ± 2	19 ± 1	23 ± 2	27 ± 1	23 ± 1	21 ± 1	-	WT ˃ KO	-	-	-
	%	0.086 ± 0.005	0.089 ± 0.007	0.070 ± 0.007	0.075 ± 0.006	0.065 ± 0.004	0.078 ± 0.003	0.078 ± 0.007	0.069 ± 0.003	Y > O *	-	-	WT: Y > O	-

Data are mean ± SEM. The number of mice: 11–12 mice per experimental condition. *: Trend (0.05 ≤ *p* < 0.1). µm^2^: mean adipocyte size. N: estimated number of adipcytes (×10^3^). %: percentage to the body weight. -: No effect. Abbreviations: Ex, exercise; KO, knockout; O, old; Sed, sedentary; *Sparc*, secreted protein acidic and rich in cysteine; WT, wild-type; Y, young. Note: No effect for the interactions Age × Exercise and Age × Genotype × Exercise (The two corresponding lines have been removed).

## Data Availability

The data presented in this study are available in article or supplementary material.

## Supplementary Materials

The following supporting information can be downloaded at: https://www.mdpi.com/article/10.3390/metabo12020125/s1, Figure S1: reports the *Sparc* Tg experiment body weights of mice.

## References

[1] Chia C.W., Egan J.M., Ferrucci L. (2018). Age-Related Changes in Glucose Metabolism, Hyperglycemia, and Cardiovascular Risk. Circ. Res..

[2] Cartee G.D. (1994). Influence of age on skeletal muscle glucose transport and glycogen metabolism. Med. Sci. Sports Exerc..

[3] Fleg J.L. (2012). Aerobic exercise in the elderly: A key to successful aging. Discov. Med..

[4] Galloza J., Castillo B., Micheo W. (2017). Benefits of Exercise in the Older Population. Phys. Med. Rehabil. Clin. N. Am..

[5] Lee P.G., Jackson E.A., Richardson C.R. (2017). Exercise Prescriptions in Older Adults. Am. Fam. Physician.

[6] Consitt L.A., Dudley C., Saxena G. (2019). Impact of Endurance and Resistance Training on Skeletal Muscle Glucose Metabolism in Older Adults. Nutrients.

[7] Riedl I., Yoshioka M., Nishida Y., Tobina T., Paradis R., Shono N., Tanaka H., St-Amand J. (2010). Regulation of skeletal muscle transcriptome in elderly men after 6 weeks of endurance training at lactate threshold intensity. Exp. Gerontol..

[8] Melouane A., Yoshioka M., Kanzaki M., St-Amand J. (2019). Sparc, an EPS-induced gene, modulates the extracellular matrix and mitochondrial function via ILK/AMPK pathways in C2C12 cells. Life Sci..

[9] Aoi W., Sakuma K. (2013). Skeletal muscle: Novel and intriguing characteristics as a secretory organ. BioDiscovery.

[10] Aoi W., Naito Y., Takagi T., Tanimura Y., Takanami Y., Kawai Y., Sakuma K., Hang L.P., Mizushima K., Hirai Y. (2013). A novel myokine, secreted protein acidic and rich in cysteine (SPARC), suppresses colon tumorigenesis via regular exercise. Gut.

[11] Ghanemi A., Melouane A., Yoshioka M., St-Amand J. (2020). Exercise Training of Secreted Protein Acidic and Rich in Cysteine (Sparc) KO Mice Suggests That Exercise-Induced Muscle Phenotype Changes Are SPARC-Dependent. Appl. Sci..

[12] Ghanemi A., Yoshioka M., St-Amand J. (2021). Measuring Exercise-Induced Secreted Protein Acidic and Rich in Cysteine Expression as a Molecular Tool to Optimize Personalized Medicine. Genes.

[13] Melouane A., Carbonell A., Yoshioka M., Puymirat J., St-Amand J. (2018). Implication of SPARC in the modulation of the extracellular matrix and mitochondrial function in muscle cells. PLoS ONE.

[14] Garneau L., Parsons S.A., Smith S.R., Mulvihill E.E., Sparks L.M., Aguer C. (2020). Plasma Myokine Concentrations After Acute Exercise in Non-obese and Obese Sedentary Women. Front. Physiol..

[15] Nakamura K., Yamanouchi K., Nishihara M. (2014). Secreted protein acidic and rich in cysteine internalization and its age-related alterations in skeletal muscle progenitor cells. Aging Cell.

[16] Delany A.M., Kalajzic I., Bradshaw A.D., Sage E.H., Canalis E. (2003). Osteonectin-null mutation compromises osteoblast formation, maturation, and survival. Endocrinology.

[17] Motamed K. (1999). SPARC (osteonectin/BM-40). Int. J. Biochem. Cell Biol..

[18] Scavelli K., Chatterjee A., Rhee D.J. (2015). Secreted Protein Acidic and Rich in Cysteine in Ocular Tissue. J. Ocul. Pharmacol. Ther..

[19] Sage H., Johnson C., Bornstein P. (1984). Characterization of a novel serum albumin-binding glycoprotein secreted by endothelial cells in culture. J. Biol. Chem..

[20] Brekken R.A., Sage E.H. (2000). SPARC, a matricellular protein: At the crossroads of cell-matrix. Matrix Biol..

[21] Norose K., Clark J.I., Syed N.A., Basu A., Heber-Katz E., Sage E.H., Howe C.C. (1998). SPARC deficiency leads to early-onset cataractogenesis. Investig. Ophthalmol. Vis. Sci..

[22] Yan Q., Sage E.H. (1999). SPARC, a matricellular glycoprotein with important biological functions. J. Histochem. Cytochem..

[23] Zhu J., Wang L.Y., Li C.Y., Wu J.Y., Zhang Y.T., Pang K.P., Wei Y., Du L.Q., Liu M., Wu X.Y. (2020). SPARC promotes self-renewal of limbal epithelial stem cells and ocular surface restoration through JNK and p38-MAPK signaling pathways. Stem Cells.

[24] Alachkar H., Santhanam R., Maharry K., Metzeler K.H., Huang X., Kohlschmidt J., Mendler J.H., Benito J.M., Hickey C., Neviani P. (2014). SPARC promotes leukemic cell growth and predicts acute myeloid leukemia outcome. J. Clin. Investig..

[25] Delany A.M., Amling M., Priemel M., Howe C., Baron R., Canalis E. (2000). Osteopenia and decreased bone formation in osteonectin-deficient mice. J. Clin. Investig..

[26] Barker T.H., Baneyx G., Cardó-Vila M., Workman G.A., Weaver M., Menon P.M., Dedhar S., Rempel S.A., Arap W., Pasqualini R. (2005). SPARC regulates extracellular matrix organization through its modulation of integrin-linked kinase activity. J. Biol. Chem..

[27] Bradshaw A.D. (2009). The role of SPARC in extracellular matrix assembly. J. Cell Commun. Signal..

[28] Ghanemi A., Melouane A., Yoshioka M., St-Amand J. (2019). Secreted protein acidic and rich in cysteine and bioenergetics: Extracellular matrix, adipocytes remodeling and skeletal muscle metabolism. Int. J. Biochem. Cell Biol..

[29] Onorato A.M., Fiore E., Bayo J., Casali C., Fernandez-Tomé M., Rodríguez M., Domínguez L., Argemi J., Hidalgo F., Favre C. (2021). SPARC inhibition accelerates NAFLD-associated hepatocellular carcinoma development by dysregulating hepatic lipid metabolism. Liver Int..

[30] Song H., Ding L., Zhang S., Wang W. (2018). MiR-29 family members interact with SPARC to regulate glucose metabolism. Biochem. Biophys. Res. Commun..

[31] Ghanemi A., Yoshioka M., St-Amand J. (2020). Secreted Protein Acidic and Rich in Cysteine: Metabolic and Homeostatic Properties beyond the Extracellular Matrix Structure. Appl. Sci..

[32] McCurdy S., Baicu C.F., Heymans S., Bradshaw A.D. (2010). Cardiac extracellular matrix remodeling: Fibrillar collagens and Secreted Protein Acidic and Rich in Cysteine (SPARC). J. Mol. Cell. Cardiol..

[33] Petersson S.J., Jørgensen L.H., Andersen D.C., Nørgaard R.C., Jensen C.H., Schrøder H.D. (2013). SPARC is up-regulated during skeletal muscle regeneration and inhibits myoblast differentiation. Histol. Histopathol..

[34] Bradshaw A.D., Sage E.H. (2001). SPARC, a matricellular protein that functions in cellular differentiation and tissue response to injury. J. Clin. Investig..

[35] Kim J.S., Galvão D.A., Newton R.U., Gray E., Taaffe D.R. (2021). Exercise-induced myokines and their effect on prostate cancer. Nat. Rev. Urol..

[36] Ghanemi A., Yoshioka M., St-Amand J. (2021). Secreted Protein Acidic and Rich in Cysteine as a Molecular Physiological and Pathological Biomarker. Biomolecules.

[37] Frontera W.R., Ochala J. (2015). Skeletal muscle: A brief review of structure and function. Calcif. Tissue Int..

[38] Barbalho S.M., Flato U.A.P., Tofano R.J., Goulart R.A., Guiguer E.L., Detregiachi C.R.P., Buchaim D.V., Araújo A.C., Buchaim R.L., Reina F.T.R. (2020). Physical Exercise and Myokines: Relationships with Sarcopenia and Cardiovascular Complications. Int. J. Mol. Sci..

[39] Lee J.H., Jun H.S. (2019). Role of Myokines in Regulating Skeletal Muscle Mass and Function. Front. Physiol..

[40] López-Otín C., Blasco M.A., Partridge L., Serrano M., Kroemer G. (2013). The hallmarks of aging. Cell.

[41] Distefano G., Goodpaster B.H. (2018). Effects of Exercise and Aging on Skeletal Muscle. Cold Spring Harb. Perspect. Med..

[42] Stegeman R., Weake V.M. (2017). Transcriptional Signatures of Aging. J. Mol. Biol..

[43] Dhingra R., Vasan R.S. (2012). Age as a risk factor. Med. Clin. N. Am..

[44] Cheitlin M.D. (2003). Cardiovascular physiology-changes with aging. Am. J. Geriatr. Cardiol..

[45] Kõks S., Dogan S., Tuna B.G., González-Navarro H., Potter P., Vandenbroucke R.E. (2016). Mouse models of ageing and their relevance to disease. Mech. Ageing Dev..

[46] Vanhooren V., Libert C. (2013). The mouse as a model organism in aging research: Usefulness, pitfalls and possibilities. Ageing Res. Rev..

[47] Azzu V., Valencak T.G. (2017). Energy Metabolism and Ageing in the Mouse: A Mini-Review. Gerontology.

[48] Azman K.F., Zakaria R. (2019). D-Galactose-induced accelerated aging model: An overview. Biogerontology.

[49] Santilli V., Bernetti A., Mangone M., Paoloni M. (2014). Clinical definition of sarcopenia. Clin. Cases Miner. Bone Metab..

[50] Jørgensen L.H., Jepsen P.L., Boysen A., Dalgaard L.B., Hvid L.G., Ørtenblad N., Ravn D., Sellathurai J., Møller-Jensen J., Lochmüller H. (2017). SPARC Interacts with Actin in Skeletal Muscle in Vitro and in Vivo. Am. J. Pathol..

[51] Kjær M. (2004). Role of Extracellular Matrix in Adaptation of Tendon and Skeletal Muscle to Mechanical Loading. Physiol. Rev..

[52] Gelse K., Pöschl E., Aigner T. (2003). Collagens—Structure, function, and biosynthesis. Adv. Drug Deliv. Rev..

[53] Wang T., Wagner A., Gehwolf R., Yan W., Passini F.S., Thien C., Weissenbacher N., Lin Z., Lehner C., Teng H. (2021). Load-induced regulation of tendon homeostasis by SPARC, a genetic predisposition factor for tendon and ligament injuries. Sci. Transl. Med..

[54] Gruber J., Schaffer S., Halliwell B. (2008). The mitochondrial free radical theory of ageing--where do we stand?. Front. Biosci..

[55] DeFronzo R.A. (1981). Glucose Intolerance and Aging. Diabetes Care.

[56] Kos K., Wong S., Tan B., Gummesson A., Jernas M., Franck N., Kerrigan D., Nystrom F.H., Carlsson L.M., Randeva H.S. (2009). Regulation of the fibrosis and angiogenesis promoter SPARC/osteonectin in human adipose tissue by weight change, leptin, insulin, and glucose. Diabetes.

[57] Lee S.H., Lee J.A., Park H.S., Song Y.S., Jang Y.J., Kim J.H., Lee Y.J., Heo Y. (2013). Associations among SPARC mRNA expression in adipose tissue, serum SPARC concentration and metabolic parameters in Korean women. Obesity.

[58] Mukherjee S., Choi M.J., Kim S.W., Yun J.W. (2020). Secreted protein acidic and rich in cysteine (SPARC) regulates thermogenesis in white and brown adipocytes. Mol. Cell. Endocrinol..

[59] Guo S.S., Zeller C., Chumlea W.C., Siervogel R.M. (1999). Aging, body composition, and lifestyle: The Fels Longitudinal Study. Am. J. Clin. Nutr..

[60] Sackmann-Sala L., Berryman D.E., Munn R.D., Lubbers E.R., Kopchick J.J. (2012). Heterogeneity among white adipose tissue depots in male C57BL/6J mice. Obesity.

[61] Jo J., Gavrilova O., Pack S., Jou W., Mullen S., Sumner A.E., Cushman S.W., Periwal V. (2009). Hypertrophy and/or Hyperplasia: Dynamics of Adipose Tissue Growth. PLoS Comput. Biol..

[62] Nie J., Bradshaw A.D., Delany A.M., Sage E.H. (2011). Inactivation of SPARC enhances high-fat diet-induced obesity in mice. Connect. Tissue Res..

[63] Carosio S., Berardinelli M.G., Aucello M., Musarò A. (2011). Impact of ageing on muscle cell regeneration. Ageing Res. Rev..

[64] Hwang A.B., Brack A.S. (2018). Muscle Stem Cells and Aging. Curr. Top. Dev. Biol..

[65] Ghanemi A., Yoshioka M., St-Amand J. (2021). Secreted Protein Acidic and Rich in Cysteine as A Regeneration Factor: Beyond the Tissue Repair. Life.

[66] Mansergh F.C., Wells T., Elford C., Evans S.L., Perry M.J., Evans M.J., Evans B.A. (2007). Osteopenia in Sparc (osteonectin)-deficient mice: Characterization of phenotypic determinants of femoral strength and changes in gene expression. Physiol. Genom..

[67] Mansergh F.C., Wride M.A., Walker V.E., Adams S., Hunter S.M., Evans M.J. (2004). Gene expression changes during cataract progression in Sparc null mice: Differential regulation of mouse globins in the lens. Mol. Vis..

[68] Rempel S.A., Hawley R.C., Gutiérrez J.A., Mouzon E., Bobbitt K.R., Lemke N., Schultz C.R., Schultz L.R., Golembieski W., Koblinski J. (2007). Splenic and immune alterations of the Sparc-null mouse accompany a lack of immune response. Genes Immun..

[69] Whittal M.C., Molladavoodi S., Zwambag D.P., Millecamps M., Stone L.S., Gregory D.E. (2021). Mechanical Consequence of Induced Intervertebral Disc Degeneration in the SPARC-Null Mouse. J. Biomech. Eng..

[70] Gruber H.E., Sage E.H., Norton H.J., Funk S., Ingram J., Hanley E.N. (2005). Targeted deletion of the SPARC gene accelerates disc degeneration in the aging mouse. J. Histochem. Cytochem..

[71] Atorrasagasti C., Onorato A., Gimeno M.L., Andreone L., Garcia M., Malvicini M., Fiore E., Bayo J., Perone M.J., Mazzolini G.D. (2019). SPARC is required for the maintenance of glucose homeostasis and insulin secretion in mice. Clin. Sci..

[72] Harries L.W., McCulloch L.J., Holley J.E., Rawling T.J., Welters H.J., Kos K. (2013). A role for SPARC in the moderation of human insulin secretion. PLoS ONE.

[73] Hu L., He F., Huang M., Zhao Q., Cheng L., Said N., Zhou Z., Liu F., Dai Y.S. (2020). SPARC promotes insulin secretion through down-regulation of RGS4 protein in pancreatic β cells. Sci. Rep..

[74] Kahn S.E., Hull R.L., Utzschneider K.M. (2006). Mechanisms linking obesity to insulin resistance and type 2 diabetes. Nature.

[75] Andrikopoulos S., Blair A.R., Deluca N., Fam B.C., Proietto J. (2008). Evaluating the glucose tolerance test in mice. Am. J. Physiol. Endocrinol. Metab..

[76] Songsorn P., Ruffino J., Vollaard N.B. (2017). No effect of acute and chronic supramaximal exercise on circulating levels of the myokine SPARC. Eur. J. Sport Sci..

[77] Batsis J.A., Villareal D.T. (2018). Sarcopenic obesity in older adults: Aetiology, epidemiology and treatment strategies. Nat. Rev. Endocrinol..

[78] Ghanemi A., Yoshioka M., St-Amand J. (2021). Obese Animals as Models for Numerous Diseases: Advantages and Applications. Medicina.

[79] Ghanemi A., Yoshioka M., St-Amand J. (2021). Ageing and Obesity Shared Patterns: From Molecular Pathogenesis to Epigenetics. Diseases.

[80] Ghanemi A., Yoshioka M., St-Amand J. (2020). Secreted protein acidic and rich in cysteine and cancer: A homeostatic hormone?. Cytokine.

[81] Aoi W., Hirano N., Lassiter D.G., Björnholm M., Chibalin A.V., Sakuma K., Tanimura Y., Mizushima K., Takagi T., Naito Y. (2019). Secreted protein acidic and rich in cysteine (SPARC) improves glucose tolerance via AMP-activated protein kinase activation. FASEB J..

[82] Richter E.A., Hargreaves M. (2013). Exercise, GLUT4, and skeletal muscle glucose uptake. Physiol. Rev..

[83] Pearson A.M. (1990). Muscle growth and exercise. Crit. Rev. Food Sci. Nutr..

[84] Liberman K., Forti L.N., Beyer I., Bautmans I. (2017). The effects of exercise on muscle strength, body composition, physical functioning and the inflammatory profile of older adults: A systematic review. Curr. Opin. Clin. Nutr. Metab. Care.

[85] Willis L.H., Slentz C.A., Bateman L.A., Shields A.T., Piner L.W., Bales C.W., Houmard J.A., Kraus W.E. (2012). Effects of aerobic and/or resistance training on body mass and fat mass in overweight or obese adults. J. Appl. Physiol..

[86] Francaux M., Deldicque L. (2019). Exercise and the control of muscle mass in human. Pflug. Arch.-Eur. J. Physiol..

[87] Bishop D.J., Botella J., Genders A.J., Lee M.J., Saner N.J., Kuang J., Yan X., Granata C. (2019). High-Intensity Exercise and Mitochondrial Biogenesis: Current Controversies and Future Research Directions. Physiology.

[88] Kwon J.H., Moon K.M., Min K.W. (2020). Exercise-Induced Myokines can Explain the Importance of Physical Activity in the Elderly: An Overview. Healthcare.

[89] Huh J.Y. (2018). The role of exercise-induced myokines in regulating metabolism. Arch. Pharmacal Res..

[90] Pedersen L., Idorn M., Olofsson G.H., Lauenborg B., Nookaew I., Hansen R.H., Johannesen H.H., Becker J.C., Pedersen K.S., Dethlefsen C. (2016). Voluntary Running Suppresses Tumor Growth through Epinephrine- and IL-6-Dependent NK Cell Mobilization and Redistribution. Cell Metab..

[91] Ghanemi A., Yoshioka M., St-Amand J. (2020). Secreted protein acidic and rich in cysteine and inflammation: Another homeostatic property?. Cytokine.

[92] Rowlatt C., Chesterman F.C., Sheriff M.U. (1976). Lifespan, age changes and tumour incidence in an ageing C57BL mouse colony. Lab. Anim..

[93] Kunstyr I., Leuenberger H.G. (1975). Gerontological data of C57BL/6J mice. I. Sex differences in survival curves. J. Gerontol..

[94] Billat V.L., Mouisel E., Roblot N., Melki J. (2005). Inter- and intrastrain variation in mouse critical running speed. J. Appl. Physiol..

[95] Schefer V., Talan M.I. (1996). Oxygen consumption in adult and AGED C57BL/6J mice during acute treadmill exercise of different intensity. Exp. Gerontol..

[96] Teklad Global 18% Protein Rodent Diet (Sterilizable). https://insights.envigo.com/hubfs/resources/data-sheets/2018s-datasheet-0915.pdf.

[97] Norose K., Lo W.K., Clark J.I., Sage E.H., Howe C.C. (2000). Lenses of SPARC-null mice exhibit an abnormal cell surface-basement membrane interface. Exp. Eye Res..

[98] Nishida Y., Tokuyama K., Nagasaka S., Higaki Y., Shirai Y., Kiyonaga A., Shindo M., Kusaka I., Nakamura T., Ishibashi S. (2004). Effect of moderate exercise training on peripheral glucose effectiveness, insulin sensitivity, and endogenous glucose production in healthy humans estimated by a two-compartment-labeled minimal model. Diabetes.

[99] Tanaka H., Shindo M. (1992). The benefits of the low intensity training. Ann. Physiol. Anthr..

[100] Motoyama M., Sunami Y., Kinoshita F., Kiyonaga A., Tanaka H., Shindo M., Irie T., Urata H., Sasaki J., Arakawa K. (1998). Blood pressure lowering effect of low intensity aerobic training in elderly hypertensive patients. Med. Sci. Sports Exerc..

[101] Sunami Y., Motoyama M., Kinoshita F., Mizooka Y., Sueta K., Matsunaga A., Sasaki J., Tanaka H., Shindo M. (1999). Effects of low-intensity aerobic training on the high-density lipoprotein cholesterol concentration in healthy elderly subjects. Metabolism.

[102] Frontera W.R., Hughes V.A., Fielding R.A., Fiatarone M.A., Evans W.J., Roubenoff R. (2000). Aging of skeletal muscle: A 12-yr longitudinal study. J. Appl. Physiol..

[103] Yamada M., Moriguch Y., Mitani T., Aoyama T., Arai H. (2014). Age-dependent changes in skeletal muscle mass and visceral fat area in Japanese adults from 40 to 79 years-of-age. Geriatr. Gerontol. Int..

[104] Miyamoto T., Shimizu Y., Matsuo Y., Otaru T., Kanzawa Y., Miyamae N., Yamada E., Katsuno T. (2021). Effects of exercise intensity and duration on a myokine, secreted protein acidic and rich in cysteine. Eur. J. Sport Sci..

[105] Potteiger J.A., Jacobsen D.J., Donnelly J.E. (2002). A comparison of methods for analyzing glucose and insulin areas under the curve following nine months of exercise in overweight adults. Int. J. Obes. Relat. Metab. Disord..

[106] Schneider C.A., Rasband W.S., Eliceiri K.W. (2012). NIH Image to ImageJ: 25 years of image analysis. Nat. Methods.

[107] Taylor S.C., Berkelman T., Yadav G., Hammond M. (2013). A defined methodology for reliable quantification of Western blot data. Mol. Biotechnol..

[108] Taylor S.C., Posch A. (2014). The design of a quantitative western blot experiment. BioMed Res. Int..

[109] Ghanemi A., Melouane A., Mucunguzi O., Yoshioka M., St-Amand J. (2018). Energy and metabolic pathways in trefoil factor family member 2 (Tff2) KO mice beyond the protection from high-fat diet-induced obesity. Life Sci..

[110] Lo S., Russell J.C., Taylor A.W. (1970). Determination of glycogen in small tissue samples. J. Appl. Physiol..

[111] Parlee S.D., Lentz S.I., Mori H., MacDougald O.A. (2014). Quantifying size and number of adipocytes in adipose tissue. Methods Enzymol..

[112] De Giorgio M.R., Yoshioka M., Riedl I., Moreault O., Cherizol R.G., Shah A.A., Blin N., Richard D., St-Amand J. (2013). Trefoil factor family member 2 (Tff2) KO mice are protected from high-fat diet-induced obesity. Obesity.

[113] https://www.cyagen.com/us/en/service/piggybac-transgenic-mouse.html.

[114] Luu-The V., Paquet N., Calvo E., Cumps J. (2005). Improved real-time RT-PCR method for high-throughput measurements using second derivative calculation and double correction. BioTechniques.

[115] Bustin S.A., Benes V., Garson J.A., Hellemans J., Huggett J., Kubista M., Mueller R., Nolan T., Pfaffl M.W., Shipley G.L. (2009). The MIQE guidelines: Minimum information for publication of quantitative real-time PCR experiments. Clin. Chem..

[116] Bustin S.A., Beaulieu J.F., Huggett J., Jaggi R., Kibenge F.S., Olsvik P.A., Penning L.C., Toegel S. (2010). MIQE précis: Practical implementation of minimum standard guidelines for fluorescence-based quantitative real-time PCR experiments. BMC Mol. Biol..

[117] Smith J.P., Hicks P.S., Ortiz L.R., Martinez M.J., Mandler R.N. (1995). Quantitative measurement of muscle strength in the mouse. J. Neurosci. Methods.

[118] Takeshita H., Yamamoto K., Nozato S., Inagaki T., Tsuchimochi H., Shirai M., Yamamoto R., Imaizumi Y., Hongyo K., Yokoyama S. (2017). Modified forelimb grip strength test detects aging-associated physiological decline in skeletal muscle function in male mice. Sci. Rep..

[119] Capaldi R.A., Malatesta F., Darley-Usmar V.M. (1983). Structure of cytochrome c oxidase. Biochim. Biophys. Acta.

[120] García-Horsman J.A., Barquera B., Rumbley J., Ma J., Gennis R.B. (1994). The superfamily of heme-copper respiratory oxidases. J. Bacteriol..

[121] Saraste M. (1994). Structure and evolution of cytochrome oxidase. Antonie Leeuwenhoek.

[122] Azzi A. (1980). Cytochrome c oxidase. Towards a clarification of its structure, interactions and mechanism. Biochim. Biophys. Acta.

[123] https://www.ncbi.nlm.nih.gov/gene/17709.

[124] Dittmer A., Dittmer J. (2006). Beta-actin is not a reliable loading control in Western blot analysis. Electrophoresis.

[125] Bao J.M., Dang Q., Lin C.J., Lo U.G., Feldkoren B., Dang A., Hernandez E., Li F., Panwar V., Lee C.F. (2021). SPARC is a key mediator of TGF-β-induced renal cancer metastasis. J. Cell. Physiol..

[126] Chang W., Wei K., Jacobs S.S., Upadhyay D., Weill D., Rosen G.D. (2010). SPARC suppresses apoptosis of idiopathic pulmonary fibrosis fibroblasts through constitutive activation of beta-catenin. J. Biol. Chem..

[127] Shen Y., Zhao Y., Yuan L., Yi W., Zhao R., Yi Q., Yong T. (2014). SPARC is over-expressed in adipose tissues of diet-induced obese rats and causes insulin resistance in 3T3-L1 adipocytes. Acta Histochem..

[128] Wu D., Li L., Yang M., Liu H., Yang G. (2011). Elevated plasma levels of SPARC in patients with newly diagnosed type 2 diabetes mellitus. Eur. J. Endocrinol..

[129] Yiu G.K., Chan W.Y., Ng S.W., Chan P.S., Cheung K.K., Berkowitz R.S., Mok S.C. (2001). SPARC (secreted protein acidic and rich in cysteine) induces apoptosis in ovarian cancer cells. Am. J. Pathol..

[130] Nakamura K., Nakano S., Miyoshi T., Yamanouchi K., Matsuwaki T., Nishihara M. (2012). Age-related resistance of skeletal muscle-derived progenitor cells to SPARC may explain a shift from myogenesis to adipogenesis. Aging.

[131] Nie J., Chang B., Traktuev D.O., Sun J., March K., Chan L., Sage E.H., Pasqualini R., Arap W., Kolonin M.G. (2008). IFATS collection: Combinatorial peptides identify alpha5beta1 integrin as a receptor for the matricellular protein SPARC on adipose stromal cells. Stem Cells.

[132] Jacunski M., Melville P., Currie G.P. (2021). Exercise: The panacea in management of many ills. Now is the time to engage. J. R. Coll. Physicians Edinb..

