# Supplementary material for: Secreted Protein Acidic and Rich in Cysteine (Sparc) KO Leads to an Accelerated Ageing Phenotype Which Is Improved by Exercise Whereas SPARC Overexpression Mimics Exercise Effects in Mice
Source: Metabolites. 2022 Jan 28;12(2):125. doi: 10.3390/metabo12020125 (PMC8879002; doi:10.3390/metabo12020125)
Supplement: Supplementary file 1 [file metabolites-12-00125-s001.zip › metabolites-1562921-SI.pdf]

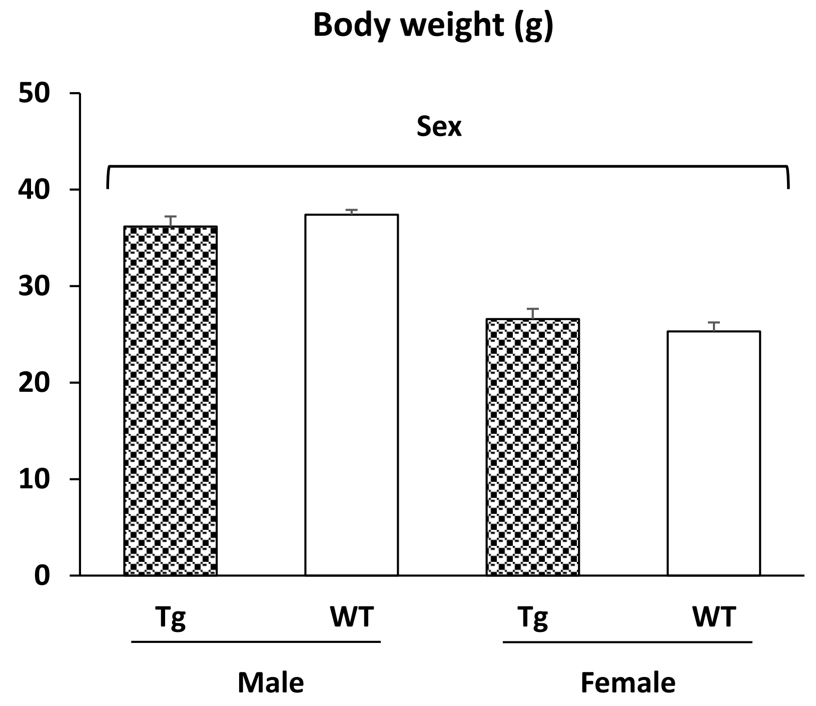

Figure S 1: Body weights of both WT and Tg mice (male and female).
Male mice had a higher body weight than female mice.
All data are mean ± SEM. The number of mice: 5-11 mice per experimental condition.
Abbreviations: g, gram; *Sparc*, secreted protein acidic and rich in cysteine; Tg, transgenic (*Sparc* overexpression); WT, wild-type.
